# Low-temperature superstructure of [(*N*,*N*-diisobutyl­carbamo­yl)meth­yl]oct­yl(phen­yl)phosphine oxide (CMPO)

**DOI:** 10.1107/S1600536811046939

**Published:** 2011-11-12

**Authors:** Michaela Pojarová, Karla Fejfarová, Emanuel Makrlík

**Affiliations:** a Institute of Physics, AS CR, v.v.i., Na Slovance 2, 182 21 Praha 8, Czech Republic; b Faculty of Environmental Sciences, Czech University of Life Sciences, Prague, Kamýcká 129, 165 21 Prague 6, Czech Republic

## Abstract

At 120 K, the title compound, C_24_H_42_NO_2_P, crystallizes in a unit cell with a doubled *a* parameter compared with the room-temperature structure. There are four mol­ecules in the asymmetric unit, one of which shows extensive disorder in a 0.588 (3):0.412 (3) ratio. In the crystal, numerous C—H⋯O inter­actions link the mol­ecules.

## Related literature

For the room-temperature structure, see: Rogers *et al.* (1995 ▶). For the use of the title compound as a ligand, see: Cherfa *et al.* (1999 ▶).
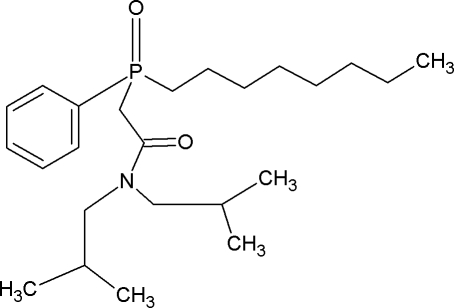


## Experimental

### 

#### Crystal data

C_24_H_42_NO_2_P
*M* *_r_* = 407.56
Monoclinic, 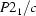

*a* = 26.1649 (3) Å
*b* = 22.0926 (3) Å
*c* = 16.9697 (2) Å
β = 90.662 (1)°
*V* = 9808.7 (2) Å^3^
*Z* = 16
Cu *K*α radiation
μ = 1.12 mm^−1^
*T* = 120 K
0.23 × 0.17 × 0.13 mm

#### Data collection

Oxford Diffraction Gemini diffractometer with an Atlas CCD detector and mirror-collimated Cu Kα radiation
Absorption correction: multi-scan (*CrysAlis PRO*; Agilent, 2009 ▶) *T*_min_ = 0.705, *T*_max_ = 1.000
79104 measured reflections
15383 independent reflections
11781 reflections with *I* > 2σ(*I*)
*R*_int_ = 0.045
θ_max_ = 62.3°

#### Refinement

*R*[*F* ^2^ > 2σ(*F* ^2^)] = 0.050
*wR*(*F* ^2^) = 0.135
*S* = 1.03
15383 reflections
1043 parameters
148 restraints
H-atom parameters constrained
Δρ_max_ = 0.80 e Å^−3^
Δρ_min_ = −0.56 e Å^−3^

### 

Data collection: *CrysAlis PRO* (Agilent, 2009 ▶); cell refinement: *CrysAlis PRO*; data reduction: *CrysAlis PRO*; program(s) used to solve structure: *SHELXS97* (Sheldrick, 2008 ▶); program(s) used to refine structure: *SHELXL97* (Sheldrick, 2008 ▶); molecular graphics: *DIAMOND* (Brandenburg & Putz, 2005 ▶); software used to prepare material for publication: *publCIF* (Westrip, 2010 ▶).

## Supplementary Material

Crystal structure: contains datablock(s) I, global. DOI: 10.1107/S1600536811046939/hb6481sup1.cif

Structure factors: contains datablock(s) I. DOI: 10.1107/S1600536811046939/hb6481Isup2.hkl

Supplementary material file. DOI: 10.1107/S1600536811046939/hb6481Isup3.cml

Additional supplementary materials:  crystallographic information; 3D view; checkCIF report

## Figures and Tables

**Table 1:** Hydrogen-bond geometry (Å, °)

*D*—H⋯*A*	*D*—H	H⋯*A*	*D*⋯*A*	*D*—H⋯*A*
C9*A*—H9*A*1⋯O2*A*^i^	0.99	2.37	3.339 (3)	165
C9*A*—H9*A*2⋯O2*D*	0.99	2.31	3.238 (5)	156
C9*B*—H9*B*2⋯O2*C*	0.99	2.49	3.442 (3)	160
C9*B*—H9*B*1⋯O2*B*^ii^	0.99	2.50	3.483 (3)	175
C9*C*—H9*C*2⋯O2*B*	0.99	2.36	3.305 (3)	158
C5*A*—H5*A*⋯O11*D*^iii^	0.95	2.48	3.379 (14)	157
C6*C*—H6*C*⋯O11*C*^iv^	0.95	2.51	3.453 (3)	170
C6*D*—H6*D*⋯O11*D*^iv^	0.95	2.47	3.37 (2)	159
C9*D*—H9*D*1⋯O11*A*^i^	0.99	2.56	3.265 (7)	128
C7*B*—H7*B*⋯O11*C*^v^	0.95	2.59	3.477 (3)	156
C9*D*—H9*D*2⋯O2*A*	0.99	2.36	3.265 (11)	151
C13*B*—H13*D*⋯O2*C*	0.99	2.52	3.431 (3)	154
C13*C*—H13*E*⋯O2*B*	0.99	2.49	3.448 (3)	162
C15*D*—H15*L*⋯O2*A*	0.98	2.55	3.332 (10)	137
C17*A*—H17*B*⋯O2*D*	0.99	2.52	3.375 (7)	145
C21*A*—H21*B*⋯O11*A*	0.99	2.52	3.139 (3)	120
C4*D*—H4*D*⋯O11*D*	0.95	2.58	3.36 (2)	140
C8*C*—H8*C*⋯O11*C*	0.95	2.52	3.239 (3)	132
C21*B*—H21*D*⋯O11*B*	0.99	2.51	3.175 (3)	124
C21*C*—H21*F*⋯O11*C*	0.99	2.43	3.093 (3)	124
C21*D*—H21*H*⋯O11*D*	0.99	2.53	3.224 (19)	127

Symmetry codes: (i) 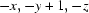; (ii) 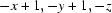; (iii) 
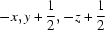
; (iv) 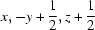; (v) 
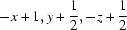
.

**Fig. 1.**
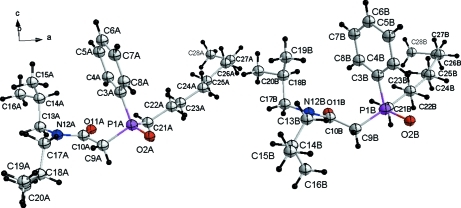
A view of first two molecules of CMPO (A,B) with atom's numbering. Displacement ellipsoids are drawn at the 50% probability level.

**Fig. 2.**
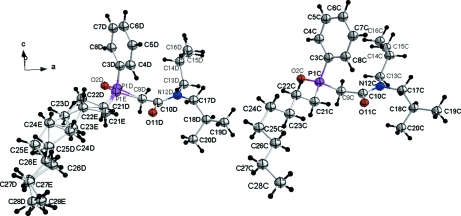
A view of second two molecules of CMPO (C,D) with atom's numbering. Displacement ellipsoids are drawn at the 50% probability level. The disordered atoms of octyl chain in molecule D were omitted for better clarity.

**Fig. 3.**
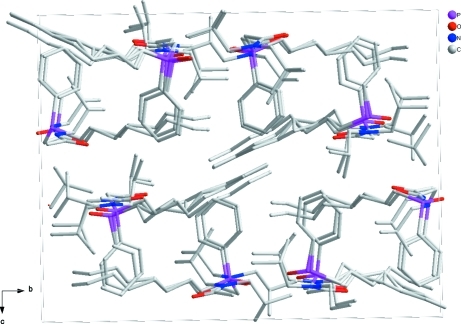
Packaging of molecules in direction of a axis.

## supplementary crystallographic information

### Comment

The ligand octyl(phenyl)-*N*,*N*-diisobutylcarbamoyl-methylphosphine oxide, C_24_H_42_N_1_O_2_P_1_, is used in extraction of transuranium elements (Cherfa *et al.*, 1999). The crystal structure of titled compound determined at room temperature (20°C) was already reported (Rogers *et al.*, 1995). The newly determined crystal structure at 120 K has also monoclinic *P*2_1_/*c* space group with a twofold length of *a* parameter which leads to four molecules (A—D) in asymmetric unit (Fig 1 and 2). The molecule D is disordered over two positions with partial occupancies 0.588 (3) and 0.412 (3). The four molecules differ in the orientation of the octyl chain. Due to the arrangement in crystal, the molecules form several types of noncovalent interactions (Table 1). They are connected *via* hydrogen bonds between the P=O group and methylene group (C9) in amide residues. There are weak intra- and intermolecular interactions between the aromatic C—H group with the amide oxygen and also intramolecular interaction between the first methylene group in octyl chain and oxygen atom in amide group.

### Experimental

Octyl(phenyl)-*N*,*N*-diisobutylcarbamoyl-methylphosphine oxide was used as received as colourless prisms from Elf-Atochem (USA).

### Refinement

The positions of disorder atoms were found from the electron density maps. Disodered fragments were then placed in appropriate positions, and all distances between neighbouring atoms were restrained as well as angles. Site occupancies were refined for the different parts with the same thermal parameters for the same atoms in various fragments. The final partial occupancies were found 0.588 (3). At the end of refinement, hydrogen atoms were placed in calculated positions with the thermal parameters *U*_iso_(H) (in the range 1.2–1.5 times *U*_eq_ of the parent atom). The electron density of terminal carbon atoms (C27 and C28) in the octyl chain was checked from maps of electron density. Based on the maps and the considerations given in details in the description of refinement, the last two terminal atoms were refined isotropically.

### Crystal data

**Table:**

C_24_H_42_NO_2_P	*F*(000) = 3584
*M**_r_* = 407.56	*D*_x_ = 1.104 Mg m^−^^3^
Monoclinic, *P*2_1_/*c*	Cu *K*α radiation, λ = 1.54180 Å
Hall symbol: -P 2ybc	Cell parameters from 37947 reflections
*a* = 26.1649 (3) Å	θ = 3.1–62.2°
*b* = 22.0926 (3) Å	µ = 1.12 mm^−^^1^
*c* = 16.9697 (2) Å	*T* = 120 K
β = 90.662 (1)°	Prism, colourless
*V* = 9808.7 (2) Å^3^	0.23 × 0.17 × 0.13 mm
*Z* = 16	

### Data collection

**Table:**

Oxford Diffraction Gemini diffractometer with an Atlas CCD detector and mirror-collimated Cu Kα radiation	15383 independent reflections
Radiation source: X-ray tube	11781 reflections with *I* > 2σ(*I*)
Mirror (Gemini ultra Cu)	*R*_int_ = 0.045
Rotation method data acquisition using ω scans	θ_max_ = 62.3°, θ_min_ = 3.3°
Absorption correction: multi-scan (*CrysAlis PRO*; Agilent, 2009)	*h* = −30→28
*T*_min_ = 0.705, *T*_max_ = 1.000	*k* = −24→25
79104 measured reflections	*l* = −19→19

### Refinement

**Table:**

Refinement on *F*^2^	Primary atom site location: structure-invariant direct methods
Least-squares matrix: full	Secondary atom site location: difference Fourier map
*R*[*F*^2^ > 2σ(*F*^2^)] = 0.050	Hydrogen site location: inferred from neighbouring sites
*wR*(*F*^2^) = 0.135	H-atom parameters constrained
*S* = 1.03	*w* = 1/[σ^2^(*F*_o_^2^) + (0.0602*P*)^2^ + 8.3042*P*] where *P* = (*F*_o_^2^ + 2*F*_c_^2^)/3
15383 reflections	(Δ/σ)_max_ = 0.001
1043 parameters	Δρ_max_ = 0.80 e Å^−^^3^
148 restraints	Δρ_min_ = −0.56 e Å^−^^3^

### Special details

**Table:**

Geometry. All e.s.d.'s (except the e.s.d. in the dihedral angle between two l.s. planes) are estimated using the full covariance matrix. The cell e.s.d.'s are taken into account individually in the estimation of e.s.d.'s in distances, angles and torsion angles; correlations between e.s.d.'s in cell parameters are only used when they are defined by crystal symmetry. An approximate (isotropic) treatment of cell e.s.d.'s is used for estimating e.s.d.'s involving l.s. planes.
Refinement. Refinement of *F*^2^ against ALL reflections. The weighted *R*-factor *wR* and goodness of fit *S* are based on *F*^2^, conventional *R*-factors *R* are based on *F*, with *F* set to zero for negative *F*^2^. The threshold expression of *F*^2^ > σ(*F*^2^) is used only for calculating *R*-factors(gt) *etc*. and is not relevant to the choice of reflections for refinement. *R*-factors based on *F*^2^ are statistically about twice as large as those based on *F*, and *R*- factors based on ALL data will be even larger. The positions of disorder atoms were found from the electron density maps. Disodered fragments were then placed in appropriate positions, and all distances between neighbouring atoms were restrained as well as angles. Site occupancies were refined for the different parts with the same thermal parameters for the same atoms in various fragments. The final partial occupancies were found 0.588 (3). At the end of refinement, hydrogen atoms were placed in calculated positions with the thermal parameters *U*_iso_(H) (in the range 1.2–1.5 times *U*_eq_ of the parent atom). The electron density of terminal carbon atoms (C27 and C28) in the octyl chain was checked from maps of electron density. Based on the maps and the considerations given in details in the description of refinement, the last two terminal atoms were refined isotropically. If refined anisotropically, the thermal ellipsoids pointed out into the C—C bonds. The difference in the *R*-factors of isotropic and anisotropic refinement was very small indicating that the wrongly oriented thermal ellipsoids did not improve the fit with experimental data. We did not find any systematic error in frame scaling or absorption correction and we also ensured the instrument stability by checking structures measured before and after this case. We finally concluded that our data set did not contain information about thermal ellipsoids of C27 and C28.

### Fractional atomic coordinates and isotropic or equivalent isotropic displacement parameters (Å^2^)

**Table:**

	*x*	*y*	*z*	*U*_iso_*/*U*_eq_	Occ. (<1)
P1A	0.01080 (2)	0.54030 (3)	0.12821 (3)	0.02449 (14)	
O2A	0.04911 (6)	0.49138 (7)	0.11735 (9)	0.0290 (4)	
O11A	−0.08727 (6)	0.61924 (7)	0.07558 (9)	0.0308 (4)	
N12A	−0.13898 (7)	0.54231 (8)	0.11059 (11)	0.0278 (4)	
C3A	−0.00385 (9)	0.55190 (10)	0.23106 (13)	0.0265 (5)	
C4A	−0.03206 (10)	0.60107 (11)	0.25772 (14)	0.0344 (6)	
H4A	−0.0440	0.6305	0.2211	0.041*	
C5A	−0.04292 (10)	0.60770 (12)	0.33718 (15)	0.0386 (6)	
H5A	−0.0623	0.6413	0.3549	0.046*	
C6A	−0.02516 (11)	0.56485 (12)	0.39031 (15)	0.0403 (6)	
H6A	−0.0322	0.5693	0.4448	0.048*	
C7A	0.00257 (11)	0.51601 (12)	0.36482 (15)	0.0436 (7)	
H7A	0.0144	0.4866	0.4017	0.052*	
C8A	0.01343 (10)	0.50941 (11)	0.28555 (14)	0.0351 (6)	
H8A	0.0328	0.4756	0.2684	0.042*	
C9A	−0.04880 (8)	0.52124 (10)	0.07875 (13)	0.0250 (5)	
H9A1	−0.0421	0.5175	0.0217	0.030*	
H9A2	−0.0598	0.4809	0.0976	0.030*	
C10A	−0.09302 (9)	0.56482 (10)	0.08903 (13)	0.0256 (5)	
C13A	−0.18165 (9)	0.58545 (11)	0.11553 (15)	0.0330 (6)	
H13A	−0.2141	0.5626	0.1118	0.040*	
H13B	−0.1803	0.6130	0.0695	0.040*	
C14A	−0.18226 (10)	0.62381 (12)	0.19074 (15)	0.0375 (6)	
H14A	−0.1522	0.6517	0.1902	0.045*	
C15A	−0.17900 (12)	0.58523 (13)	0.26482 (16)	0.0464 (7)	
H15A	−0.2084	0.5579	0.2665	0.056*	
H15B	−0.1791	0.6115	0.3113	0.056*	
H15C	−0.1474	0.5614	0.2644	0.056*	
C16A	−0.23083 (11)	0.66192 (14)	0.18990 (18)	0.0515 (8)	
H16A	−0.2328	0.6852	0.1408	0.062*	
H16B	−0.2302	0.6898	0.2348	0.062*	
H16C	−0.2607	0.6353	0.1935	0.062*	
C17A	−0.14954 (9)	0.47810 (10)	0.12517 (14)	0.0293 (5)	
H17A	−0.1800	0.4750	0.1591	0.035*	
H17B	−0.1203	0.4605	0.1547	0.035*	
C18A	−0.15897 (10)	0.43999 (12)	0.05064 (15)	0.0354 (6)	
H18A	−0.1264	0.4389	0.0203	0.042*	
C19A	−0.17116 (11)	0.37578 (12)	0.07612 (19)	0.0481 (7)	
H19A	−0.2025	0.3758	0.1072	0.058*	
H19B	−0.1428	0.3599	0.1082	0.058*	
H19C	−0.1760	0.3502	0.0294	0.058*	
C20A	−0.20040 (11)	0.46545 (14)	−0.00288 (16)	0.0473 (7)	
H20A	−0.2034	0.4404	−0.0504	0.057*	
H20B	−0.1916	0.5070	−0.0177	0.057*	
H20C	−0.2330	0.4654	0.0249	0.057*	
C21A	0.03224 (9)	0.61198 (10)	0.09086 (13)	0.0269 (5)	
H21A	0.0365	0.6088	0.0331	0.032*	
H21B	0.0057	0.6429	0.1009	0.032*	
C22A	0.08282 (9)	0.63280 (10)	0.12820 (14)	0.0282 (5)	
H22A	0.0803	0.6305	0.1863	0.034*	
H22B	0.1105	0.6053	0.1116	0.034*	
C23A	0.09604 (9)	0.69722 (11)	0.10437 (14)	0.0316 (6)	
H23A	0.0983	0.6991	0.0462	0.038*	
H23B	0.0679	0.7243	0.1205	0.038*	
C24A	0.14580 (9)	0.72052 (11)	0.13988 (15)	0.0314 (6)	
H24A	0.1748	0.6996	0.1146	0.038*	
H24B	0.1469	0.7107	0.1968	0.038*	
C25A	0.15209 (10)	0.78869 (11)	0.12953 (15)	0.0355 (6)	
H25A	0.1568	0.7975	0.0729	0.043*	
H25B	0.1202	0.8089	0.1462	0.043*	
C26A	0.19661 (11)	0.81547 (13)	0.17551 (17)	0.0457 (7)	
H26A	0.2279	0.7920	0.1639	0.055*	
H26B	0.1899	0.8116	0.2326	0.055*	
C27A	0.20618 (13)	0.88229 (15)	0.1562 (2)	0.0631 (9)*	
H27A	0.2331	0.8980	0.1922	0.076*	
H27B	0.2193	0.8852	0.1018	0.076*	
C28A	0.15983 (15)	0.92178 (18)	0.1628 (2)	0.0764 (11)*	
H28A	0.1347	0.9103	0.1222	0.092*	
H28B	0.1697	0.9642	0.1554	0.092*	
H28C	0.1448	0.9167	0.2150	0.092*	
P1B	0.50464 (2)	0.52944 (3)	0.13204 (3)	0.02519 (14)	
O2B	0.54306 (6)	0.48119 (7)	0.11856 (10)	0.0312 (4)	
O11B	0.40211 (6)	0.60593 (7)	0.08700 (9)	0.0322 (4)	
N12B	0.35704 (7)	0.52836 (9)	0.13750 (12)	0.0311 (5)	
C3B	0.49258 (9)	0.54066 (10)	0.23595 (13)	0.0262 (5)	
C4B	0.51457 (10)	0.50018 (11)	0.28938 (14)	0.0336 (6)	
H4B	0.5345	0.4673	0.2709	0.040*	
C5B	0.50736 (11)	0.50791 (13)	0.36968 (15)	0.0426 (7)	
H5B	0.5226	0.4804	0.4060	0.051*	
C6B	0.47839 (10)	0.55507 (12)	0.39685 (15)	0.0389 (6)	
H6B	0.4734	0.5598	0.4519	0.047*	
C7B	0.45637 (10)	0.59593 (12)	0.34453 (14)	0.0356 (6)	
H7B	0.4365	0.6287	0.3635	0.043*	
C8B	0.46360 (9)	0.58847 (11)	0.26407 (14)	0.0305 (5)	
H8B	0.4486	0.6163	0.2280	0.037*	
C9B	0.44415 (9)	0.51036 (10)	0.08521 (13)	0.0271 (5)	
H9B1	0.4490	0.5103	0.0274	0.032*	
H9B2	0.4349	0.4686	0.1009	0.032*	
C10B	0.39946 (9)	0.55158 (11)	0.10363 (13)	0.0275 (5)	
C13B	0.34851 (10)	0.46423 (11)	0.15607 (15)	0.0336 (6)	
H13C	0.3316	0.4614	0.2078	0.057 (9)*	
H13D	0.3820	0.4437	0.1609	0.038 (7)*	
C14B	0.31632 (12)	0.43117 (13)	0.09542 (18)	0.0484 (7)	
H14B	0.2819	0.4507	0.0928	0.058*	
C15B	0.30975 (13)	0.36567 (14)	0.1228 (2)	0.0647 (10)	
H15D	0.2870	0.3442	0.0859	0.078*	
H15E	0.2949	0.3652	0.1755	0.078*	
H15F	0.3431	0.3456	0.1244	0.078*	
C16B	0.33887 (14)	0.43268 (15)	0.01548 (19)	0.0617 (9)	
H16D	0.3449	0.4748	0.0000	0.074*	
H16E	0.3152	0.4137	−0.0223	0.074*	
H16F	0.3713	0.4106	0.0160	0.074*	
C17B	0.31320 (10)	0.56925 (12)	0.14730 (16)	0.0362 (6)	
H17C	0.2817	0.5446	0.1491	0.043*	
H17D	0.3106	0.5957	0.1003	0.043*	
C18B	0.31535 (11)	0.60941 (13)	0.22088 (16)	0.0425 (7)	
H18B	0.3454	0.6372	0.2166	0.051*	
C19B	0.32103 (13)	0.57310 (15)	0.29629 (17)	0.0542 (8)	
H19D	0.3229	0.6007	0.3414	0.065*	
H19E	0.3524	0.5489	0.2943	0.065*	
H19F	0.2915	0.5462	0.3019	0.065*	
C20B	0.26669 (12)	0.64766 (15)	0.2222 (2)	0.0581 (8)	
H20D	0.2369	0.6211	0.2279	0.070*	
H20E	0.2635	0.6704	0.1728	0.070*	
H20F	0.2684	0.6759	0.2667	0.070*	
C21B	0.52336 (9)	0.60184 (10)	0.09378 (13)	0.0267 (5)	
H21C	0.5284	0.5981	0.0362	0.032*	
H21D	0.4953	0.6311	0.1022	0.032*	
C22B	0.57236 (9)	0.62703 (10)	0.13147 (14)	0.0301 (5)	
H22C	0.5676	0.6315	0.1890	0.036*	
H22D	0.6008	0.5982	0.1231	0.036*	
C23B	0.58611 (10)	0.68828 (11)	0.09612 (15)	0.0323 (6)	
H23C	0.5923	0.6829	0.0391	0.039*	
H23D	0.5565	0.7158	0.1016	0.039*	
C24B	0.63263 (11)	0.71789 (12)	0.13350 (16)	0.0399 (6)	
H24C	0.6633	0.6935	0.1216	0.048*	
H24D	0.6286	0.7186	0.1914	0.048*	
C25B	0.64055 (11)	0.78268 (12)	0.10373 (16)	0.0416 (7)	
H25C	0.6534	0.7809	0.0491	0.050*	
H25D	0.6071	0.8035	0.1022	0.050*	
C26B	0.67734 (13)	0.81975 (13)	0.15320 (18)	0.0534 (8)	
H26C	0.7110	0.7993	0.1540	0.064*	
H26D	0.6648	0.8210	0.2080	0.064*	
C27B	0.68431 (12)	0.88457 (14)	0.12384 (19)	0.0537 (8)*	
H27C	0.7090	0.9055	0.1591	0.064*	
H27D	0.6994	0.8832	0.0706	0.064*	
C28B	0.63552 (12)	0.92151 (15)	0.11984 (19)	0.0573 (8)*	
H28D	0.6121	0.9036	0.0809	0.069*	
H28E	0.6436	0.9631	0.1043	0.069*	
H28F	0.6194	0.9217	0.1717	0.069*	
P1C	0.48393 (2)	0.31171 (3)	0.14073 (4)	0.02857 (15)	
O2C	0.44377 (6)	0.35912 (7)	0.13383 (10)	0.0357 (4)	
O11C	0.58035 (7)	0.23781 (7)	0.08795 (10)	0.0349 (4)	
N12C	0.63308 (8)	0.31775 (9)	0.10350 (11)	0.0305 (5)	
C3C	0.50522 (9)	0.30133 (10)	0.24182 (14)	0.0289 (5)	
C4C	0.48844 (10)	0.34266 (11)	0.29777 (15)	0.0358 (6)	
H4C	0.4658	0.3743	0.2827	0.043*	
C5C	0.50478 (11)	0.33764 (13)	0.37570 (16)	0.0428 (7)	
H5C	0.4931	0.3659	0.4137	0.051*	
C6C	0.53789 (11)	0.29179 (12)	0.39841 (15)	0.0405 (7)	
H6C	0.5494	0.2890	0.4516	0.049*	
C7C	0.55427 (10)	0.24977 (12)	0.34312 (15)	0.0374 (6)	
H7C	0.5765	0.2178	0.3586	0.045*	
C8C	0.53805 (10)	0.25472 (11)	0.26523 (14)	0.0331 (6)	
H8C	0.5494	0.2261	0.2275	0.040*	
C9C	0.53970 (9)	0.33385 (11)	0.08537 (14)	0.0303 (5)	
H9C1	0.5298	0.3359	0.0289	0.036*	
H9C2	0.5496	0.3752	0.1020	0.036*	
C10C	0.58629 (9)	0.29334 (11)	0.09318 (13)	0.0279 (5)	
C13C	0.64498 (10)	0.38276 (11)	0.10708 (15)	0.0335 (6)	
H13E	0.6129	0.4060	0.1002	0.040*	
H13F	0.6676	0.3931	0.0627	0.040*	
C14C	0.67081 (10)	0.40213 (12)	0.18392 (15)	0.0374 (6)	
H14C	0.7055	0.3833	0.1861	0.045*	
C15C	0.67757 (12)	0.47057 (12)	0.18362 (17)	0.0473 (7)	
H15G	0.6439	0.4901	0.1819	0.057*	
H15H	0.6971	0.4826	0.1373	0.057*	
H15I	0.6960	0.4831	0.2315	0.057*	
C16C	0.64203 (13)	0.38139 (15)	0.25495 (17)	0.0561 (8)	
H16G	0.6072	0.3978	0.2529	0.067*	
H16H	0.6595	0.3958	0.3028	0.067*	
H16I	0.6406	0.3371	0.2555	0.067*	
C17C	0.67684 (10)	0.27641 (11)	0.10617 (15)	0.0347 (6)	
H17E	0.6644	0.2351	0.1182	0.042*	
H17F	0.7001	0.2889	0.1496	0.042*	
C18C	0.70634 (11)	0.27449 (15)	0.03107 (19)	0.0533 (8)	
H18C	0.7224	0.3151	0.0238	0.064*	
C19C	0.74963 (13)	0.22836 (18)	0.0419 (2)	0.0687 (10)	
H19G	0.7351	0.1876	0.0460	0.082*	
H19H	0.7690	0.2378	0.0901	0.082*	
H19I	0.7724	0.2302	−0.0034	0.082*	
C20C	0.67531 (13)	0.26156 (16)	−0.03997 (18)	0.0617 (9)	
H20G	0.6582	0.2224	−0.0339	0.074*	
H20H	0.6975	0.2603	−0.0861	0.074*	
H20I	0.6496	0.2934	−0.0472	0.074*	
C21C	0.46282 (10)	0.23899 (11)	0.10529 (14)	0.0314 (6)	
H21E	0.4529	0.2427	0.0490	0.038*	
H21F	0.4917	0.2101	0.1089	0.038*	
C22C	0.41774 (10)	0.21348 (11)	0.15081 (15)	0.0343 (6)	
H22E	0.4289	0.2052	0.2057	0.041*	
H22F	0.3904	0.2444	0.1526	0.041*	
C23C	0.39600 (10)	0.15571 (11)	0.11521 (15)	0.0339 (6)	
H23E	0.4238	0.1255	0.1110	0.041*	
H23F	0.3833	0.1645	0.0612	0.041*	
C24C	0.35265 (10)	0.12820 (11)	0.16242 (15)	0.0353 (6)	
H24E	0.3289	0.1609	0.1779	0.042*	
H24F	0.3671	0.1104	0.2114	0.042*	
C25C	0.32236 (10)	0.07946 (11)	0.11835 (15)	0.0362 (6)	
H25E	0.2945	0.0651	0.1527	0.043*	
H25F	0.3063	0.0980	0.0711	0.043*	
C26C	0.35367 (10)	0.02525 (11)	0.09268 (15)	0.0340 (6)	
H26E	0.3803	0.0391	0.0559	0.041*	
H26F	0.3712	0.0078	0.1395	0.041*	
C27C	0.32204 (10)	−0.02377 (12)	0.05281 (16)	0.0398 (6)*	
H27E	0.3025	−0.0056	0.0084	0.048*	
H27F	0.2971	−0.0399	0.0910	0.048*	
C28C	0.35400 (12)	−0.07582 (13)	0.02177 (18)	0.0495 (7)*	
H28G	0.3770	−0.0608	−0.0190	0.059*	
H28H	0.3314	−0.1069	−0.0008	0.059*	
H28I	0.3742	−0.0934	0.0651	0.059*	
P1D	−0.0132 (2)	0.3322 (2)	0.1553 (2)	0.0261 (4)	0.588 (3)
O2D	−0.0483 (3)	0.3850 (2)	0.1491 (4)	0.0272 (11)	0.588 (3)
O11D	0.0778 (7)	0.2446 (5)	0.0928 (13)	0.028 (3)	0.588 (3)
N12D	0.1349 (4)	0.3187 (8)	0.1196 (16)	0.0264 (5)	0.588 (3)
C3D	0.0067 (2)	0.3186 (2)	0.25637 (19)	0.0302 (8)	0.588 (3)
C4D	0.0390 (2)	0.2705 (2)	0.2779 (2)	0.0302 (8)	0.588 (3)
H4D	0.0514	0.2437	0.2388	0.036*	0.588 (3)
C5D	0.05270 (15)	0.26211 (17)	0.3567 (3)	0.0302 (8)	0.588 (3)
H5D	0.0749	0.2298	0.3711	0.036*	0.588 (3)
C6D	0.03407 (13)	0.30070 (17)	0.4146 (2)	0.0302 (8)	0.588 (3)
H6D	0.0477	0.2991	0.4667	0.036*	0.588 (3)
C7D	−0.00539 (13)	0.34242 (15)	0.3951 (2)	0.0302 (8)	0.588 (3)
H7D	−0.0262	0.3598	0.4346	0.036*	0.588 (3)
C8D	−0.01251 (14)	0.35727 (15)	0.3137 (2)	0.0302 (8)	0.588 (3)
H8D	−0.0301	0.3932	0.2988	0.036*	0.588 (3)
C9D	0.0440 (3)	0.3445 (5)	0.0978 (4)	0.0265 (6)	0.588 (3)
H9D1	0.0340	0.3451	0.0413	0.032*	0.588 (3)
H9D2	0.0577	0.3851	0.1110	0.032*	0.588 (3)
C10D	0.0866 (4)	0.2986 (5)	0.1088 (13)	0.0254 (9)	0.588 (3)
C13D	0.1491 (5)	0.3824 (9)	0.1321 (10)	0.0275 (7)	0.588 (3)
H13G	0.1278	0.4079	0.0967	0.033*	0.588 (3)
H13H	0.1852	0.3878	0.1167	0.033*	0.588 (3)
C14D	0.1429 (5)	0.4049 (6)	0.2168 (8)	0.0364 (11)	0.588 (3)
H14D	0.1058	0.4020	0.2300	0.044*	0.588 (3)
C15D	0.1576 (3)	0.4723 (5)	0.2191 (8)	0.0362 (18)	0.588 (3)
H15J	0.1944	0.4764	0.2101	0.043*	0.588 (3)
H15K	0.1492	0.4892	0.2707	0.043*	0.588 (3)
H15L	0.1386	0.4941	0.1778	0.043*	0.588 (3)
C16D	0.1724 (9)	0.3678 (8)	0.2773 (12)	0.048 (3)	0.588 (3)
H16J	0.1598	0.3260	0.2767	0.057*	0.588 (3)
H16K	0.1676	0.3852	0.3298	0.057*	0.588 (3)
H16L	0.2088	0.3682	0.2645	0.057*	0.588 (3)
C17D	0.1761 (6)	0.2736 (10)	0.1250 (10)	0.0305 (9)	0.588 (3)
H17G	0.1615	0.2349	0.1438	0.037*	0.588 (3)
H17H	0.2015	0.2873	0.1649	0.037*	0.588 (3)
C18D	0.2034 (6)	0.2622 (7)	0.0476 (10)	0.0307 (13)	0.588 (3)
H18D	0.2194	0.3011	0.0306	0.037*	0.588 (3)
C19D	0.2460 (8)	0.2166 (13)	0.0631 (12)	0.040 (3)	0.588 (3)
H19J	0.2693	0.2324	0.1038	0.048*	0.588 (3)
H19K	0.2650	0.2097	0.0144	0.048*	0.588 (3)
H19L	0.2312	0.1783	0.0810	0.048*	0.588 (3)
C20D	0.1675 (9)	0.2410 (10)	−0.0183 (10)	0.037 (2)	0.588 (3)
H20J	0.1491	0.2047	−0.0011	0.044*	0.588 (3)
H20K	0.1874	0.2315	−0.0653	0.044*	0.588 (3)
H20L	0.1428	0.2731	−0.0307	0.044*	0.588 (3)
C21D	−0.0428 (2)	0.2629 (3)	0.1215 (4)	0.0257 (12)	0.588 (3)
H21G	−0.0566	0.2690	0.0675	0.031*	0.588 (3)
H21H	−0.0166	0.2306	0.1192	0.031*	0.588 (3)
C22D	−0.0860 (2)	0.2427 (2)	0.1755 (3)	0.0288 (11)	0.588 (3)
H22G	−0.0712	0.2206	0.2212	0.035*	0.588 (3)
H22H	−0.1038	0.2789	0.1959	0.035*	0.588 (3)
C23D	−0.12469 (15)	0.20207 (18)	0.1340 (2)	0.0312 (8)	0.588 (3)
H23G	−0.1369	0.2229	0.0857	0.037*	0.588 (3)
H23H	−0.1545	0.1968	0.1688	0.037*	0.588 (3)
C24D	−0.10496 (16)	0.13974 (19)	0.1111 (3)	0.0322 (8)	0.588 (3)
H24G	−0.0873	0.1212	0.1571	0.039*	0.588 (3)
H24H	−0.0798	0.1441	0.0684	0.039*	0.588 (3)
C25D	−0.14814 (18)	0.09821 (19)	0.0835 (3)	0.0353 (10)	0.588 (3)
H25G	−0.1724	0.0933	0.1273	0.042*	0.588 (3)
H25H	−0.1667	0.1185	0.0397	0.042*	0.588 (3)
C26D	−0.1322 (3)	0.0359 (4)	0.0559 (9)	0.0372 (18)	0.588 (3)
H26G	−0.1120	0.0161	0.0983	0.045*	0.588 (3)
H26H	−0.1098	0.0403	0.0096	0.045*	0.588 (3)
C27D	−0.1772 (4)	−0.0046 (11)	0.034 (3)	0.0427 (7)	0.588 (3)
H27G	−0.1970	−0.0135	0.0817	0.051*	0.588 (3)
H27H	−0.1999	0.0175	−0.0034	0.051*	0.588 (3)
C28D	−0.1614 (4)	−0.0640 (6)	−0.0041 (14)	0.054 (3)	0.588 (3)
H28J	−0.1420	−0.0557	−0.0519	0.065*	0.588 (3)
H28K	−0.1920	−0.0875	−0.0178	0.065*	0.588 (3)
H28L	−0.1401	−0.0871	0.0330	0.065*	0.588 (3)
P1E	−0.0150 (3)	0.3228 (3)	0.1529 (3)	0.0261 (4)	0.412 (3)
O2E	−0.0548 (4)	0.3703 (4)	0.1425 (7)	0.0272 (11)	0.412 (3)
O11E	0.0751 (10)	0.2442 (7)	0.107 (2)	0.028 (3)	0.412 (3)
N12E	0.1344 (6)	0.3177 (11)	0.121 (2)	0.0264 (5)	0.412 (3)
C3E	0.0011 (3)	0.3118 (3)	0.2561 (3)	0.0326 (12)	0.412 (3)
C4E	0.0334 (3)	0.2651 (3)	0.2820 (4)	0.0326 (12)	0.412 (3)
H4E	0.0486	0.2386	0.2449	0.039*	0.412 (3)
C5E	0.0434 (2)	0.2573 (3)	0.3625 (4)	0.0326 (12)	0.412 (3)
H5E	0.0639	0.2245	0.3803	0.039*	0.412 (3)
C6E	0.02312 (18)	0.2980 (3)	0.4167 (3)	0.0326 (12)	0.412 (3)
H6E	0.0241	0.2892	0.4715	0.039*	0.412 (3)
C7E	0.00095 (19)	0.3530 (2)	0.3890 (3)	0.0326 (12)	0.412 (3)
H7E	0.0001	0.3885	0.4205	0.039*	0.412 (3)
C8E	−0.0201 (2)	0.3516 (2)	0.3105 (3)	0.0326 (12)	0.412 (3)
H8E	−0.0478	0.3772	0.2960	0.039*	0.412 (3)
C9E	0.0434 (4)	0.3452 (7)	0.1035 (6)	0.0265 (6)	0.412 (3)
H9E1	0.0348	0.3548	0.0479	0.032*	0.412 (3)
H9E2	0.0563	0.3828	0.1284	0.032*	0.412 (3)
C10E	0.0863 (5)	0.2990 (7)	0.1044 (19)	0.0254 (9)	0.412 (3)
C13E	0.1499 (7)	0.3815 (12)	0.1271 (15)	0.0275 (7)	0.412 (3)
H13I	0.1293	0.4056	0.0893	0.033*	0.412 (3)
H13J	0.1861	0.3850	0.1116	0.033*	0.412 (3)
C14E	0.1439 (7)	0.4090 (9)	0.2095 (12)	0.0364 (11)	0.412 (3)
H14E	0.1067	0.4165	0.2177	0.044*	0.412 (3)
C15E	0.1711 (6)	0.4707 (9)	0.2103 (13)	0.0362 (18)	0.412 (3)
H15M	0.2068	0.4654	0.1951	0.043*	0.412 (3)
H15N	0.1697	0.4880	0.2633	0.043*	0.412 (3)
H15O	0.1540	0.4980	0.1729	0.043*	0.412 (3)
C16E	0.1626 (14)	0.3678 (12)	0.2755 (17)	0.048 (3)	0.412 (3)
H16M	0.1470	0.3277	0.2694	0.057*	0.412 (3)
H16N	0.1529	0.3851	0.3264	0.057*	0.412 (3)
H16O	0.1998	0.3641	0.2733	0.057*	0.412 (3)
C17E	0.1756 (8)	0.2724 (15)	0.1187 (14)	0.0305 (9)	0.412 (3)
H17I	0.1612	0.2325	0.1330	0.037*	0.412 (3)
H17J	0.2016	0.2830	0.1591	0.037*	0.412 (3)
C18E	0.2016 (8)	0.2668 (11)	0.0392 (15)	0.0307 (13)	0.412 (3)
H18E	0.2167	0.3071	0.0259	0.037*	0.412 (3)
C19E	0.2453 (12)	0.222 (2)	0.0478 (18)	0.040 (3)	0.412 (3)
H19M	0.2693	0.2357	0.0885	0.048*	0.412 (3)
H19N	0.2630	0.2179	−0.0025	0.048*	0.412 (3)
H19O	0.2315	0.1820	0.0629	0.048*	0.412 (3)
C20E	0.1649 (13)	0.2491 (16)	−0.0273 (14)	0.037 (2)	0.412 (3)
H20M	0.1500	0.2093	−0.0160	0.044*	0.412 (3)
H20N	0.1834	0.2472	−0.0771	0.044*	0.412 (3)
H20O	0.1375	0.2792	−0.0316	0.044*	0.412 (3)
C21E	−0.0339 (3)	0.2503 (4)	0.1138 (5)	0.0257 (12)	0.412 (3)
H21I	−0.0390	0.2541	0.0561	0.031*	0.412 (3)
H21J	−0.0058	0.2211	0.1230	0.031*	0.412 (3)
C22E	−0.0827 (3)	0.2248 (3)	0.1494 (4)	0.0288 (11)	0.412 (3)
H22I	−0.0757	0.2137	0.2050	0.035*	0.412 (3)
H22J	−0.1092	0.2569	0.1490	0.035*	0.412 (3)
C23E	−0.1036 (2)	0.1696 (3)	0.1061 (4)	0.0312 (8)	0.412 (3)
H23I	−0.1157	0.1822	0.0531	0.037*	0.412 (3)
H23J	−0.0755	0.1402	0.0991	0.037*	0.412 (3)
C24E	−0.1474 (2)	0.1383 (2)	0.1481 (3)	0.0322 (8)	0.412 (3)
H24I	−0.1335	0.1177	0.1956	0.039*	0.412 (3)
H24J	−0.1718	0.1694	0.1660	0.039*	0.412 (3)
C25E	−0.1762 (3)	0.0921 (3)	0.0983 (4)	0.0353 (10)	0.412 (3)
H25I	−0.2046	0.0756	0.1299	0.042*	0.412 (3)
H25J	−0.1916	0.1132	0.0523	0.042*	0.412 (3)
C26E	−0.1447 (4)	0.0395 (6)	0.0683 (14)	0.0372 (18)	0.412 (3)
H26I	−0.1250	0.0220	0.1129	0.045*	0.412 (3)
H26J	−0.1199	0.0549	0.0294	0.045*	0.412 (3)
C27E	−0.1767 (5)	−0.0102 (15)	0.030 (4)	0.0427 (7)	0.412 (3)
H27I	−0.2013	−0.0257	0.0691	0.051*	0.412 (3)
H27J	−0.1966	0.0074	−0.0143	0.051*	0.412 (3)
C28E	−0.1452 (7)	−0.0627 (8)	−0.001 (2)	0.054 (3)	0.412 (3)
H28M	−0.1241	−0.0489	−0.0444	0.065*	0.412 (3)
H28N	−0.1681	−0.0950	−0.0190	0.065*	0.412 (3)
H28O	−0.1231	−0.0782	0.0418	0.065*	0.412 (3)

### Atomic displacement parameters (Å^2^)

**Table:**

	*U*^11^	*U*^22^	*U*^33^	*U*^12^	*U*^13^	*U*^23^
P1A	0.0258 (3)	0.0229 (3)	0.0247 (3)	0.0009 (2)	−0.0012 (2)	−0.0019 (2)
O2A	0.0275 (9)	0.0260 (8)	0.0336 (9)	0.0040 (7)	−0.0010 (7)	−0.0040 (7)
O11A	0.0327 (10)	0.0265 (9)	0.0330 (9)	0.0028 (7)	0.0011 (7)	0.0011 (7)
N12A	0.0271 (11)	0.0258 (10)	0.0305 (10)	0.0028 (8)	0.0001 (8)	−0.0015 (8)
C3A	0.0258 (13)	0.0257 (12)	0.0281 (12)	−0.0029 (10)	−0.0018 (10)	−0.0031 (10)
C4A	0.0407 (15)	0.0318 (13)	0.0307 (13)	0.0040 (12)	−0.0030 (11)	−0.0026 (11)
C5A	0.0459 (16)	0.0373 (15)	0.0326 (14)	0.0029 (12)	0.0011 (12)	−0.0095 (11)
C6A	0.0502 (17)	0.0444 (16)	0.0264 (13)	−0.0103 (13)	0.0013 (12)	−0.0047 (12)
C7A	0.0598 (19)	0.0385 (15)	0.0324 (14)	0.0010 (14)	−0.0022 (13)	0.0058 (12)
C8A	0.0443 (16)	0.0297 (13)	0.0312 (13)	0.0022 (12)	−0.0006 (11)	0.0015 (10)
C9A	0.0260 (12)	0.0246 (12)	0.0244 (12)	0.0003 (10)	0.0011 (10)	−0.0024 (9)
C10A	0.0284 (13)	0.0285 (13)	0.0199 (11)	0.0001 (10)	−0.0016 (9)	−0.0032 (9)
C13A	0.0271 (13)	0.0342 (14)	0.0378 (14)	0.0051 (11)	0.0008 (11)	0.0004 (11)
C14A	0.0363 (15)	0.0356 (14)	0.0408 (15)	0.0067 (12)	0.0069 (12)	−0.0028 (11)
C15A	0.0517 (18)	0.0506 (17)	0.0370 (15)	0.0082 (14)	0.0067 (13)	−0.0057 (13)
C16A	0.0494 (18)	0.0503 (18)	0.0552 (18)	0.0203 (15)	0.0129 (15)	−0.0001 (14)
C17A	0.0275 (13)	0.0295 (13)	0.0309 (13)	−0.0012 (10)	0.0018 (10)	−0.0006 (10)
C18A	0.0293 (14)	0.0383 (14)	0.0386 (14)	−0.0045 (11)	−0.0007 (11)	−0.0068 (11)
C19A	0.0409 (17)	0.0383 (16)	0.0649 (19)	−0.0053 (13)	−0.0072 (14)	−0.0110 (14)
C20A	0.0462 (17)	0.0553 (18)	0.0401 (15)	−0.0056 (14)	−0.0079 (13)	−0.0055 (13)
C21A	0.0272 (13)	0.0268 (12)	0.0267 (12)	0.0023 (10)	−0.0013 (10)	−0.0007 (9)
C22A	0.0287 (13)	0.0256 (12)	0.0303 (12)	0.0012 (10)	−0.0022 (10)	0.0001 (10)
C23A	0.0306 (14)	0.0297 (13)	0.0344 (13)	−0.0005 (11)	−0.0009 (11)	0.0039 (10)
C24A	0.0302 (14)	0.0300 (13)	0.0340 (13)	−0.0012 (11)	0.0001 (11)	0.0003 (10)
C25A	0.0360 (15)	0.0316 (14)	0.0391 (14)	−0.0026 (11)	0.0042 (12)	−0.0003 (11)
C26A	0.0516 (18)	0.0434 (16)	0.0420 (15)	−0.0174 (14)	−0.0036 (13)	0.0011 (13)
P1B	0.0280 (3)	0.0217 (3)	0.0259 (3)	0.0019 (2)	−0.0002 (2)	−0.0004 (2)
O2B	0.0300 (9)	0.0249 (8)	0.0387 (9)	0.0043 (7)	0.0032 (7)	−0.0015 (7)
O11B	0.0348 (10)	0.0281 (9)	0.0334 (9)	0.0028 (7)	−0.0039 (7)	0.0000 (7)
N12B	0.0270 (11)	0.0298 (11)	0.0363 (11)	0.0023 (9)	−0.0039 (9)	−0.0010 (9)
C3B	0.0245 (12)	0.0252 (12)	0.0289 (12)	−0.0035 (10)	−0.0002 (10)	0.0014 (10)
C4B	0.0342 (14)	0.0306 (13)	0.0360 (14)	0.0007 (11)	−0.0002 (11)	0.0081 (11)
C5B	0.0473 (17)	0.0472 (16)	0.0332 (14)	−0.0002 (14)	−0.0071 (12)	0.0153 (12)
C6B	0.0443 (16)	0.0474 (16)	0.0250 (13)	−0.0105 (13)	−0.0003 (11)	0.0020 (12)
C7B	0.0413 (15)	0.0347 (14)	0.0308 (13)	−0.0035 (12)	0.0012 (11)	−0.0050 (11)
C8B	0.0351 (14)	0.0289 (13)	0.0274 (12)	0.0009 (11)	−0.0041 (11)	−0.0006 (10)
C9B	0.0289 (13)	0.0267 (12)	0.0256 (12)	−0.0011 (10)	−0.0002 (10)	−0.0030 (9)
C10B	0.0302 (13)	0.0291 (13)	0.0231 (12)	0.0002 (10)	−0.0056 (10)	−0.0028 (10)
C13B	0.0297 (14)	0.0337 (14)	0.0374 (14)	−0.0003 (11)	−0.0033 (11)	0.0040 (11)
C14B	0.0489 (18)	0.0399 (16)	0.0562 (18)	−0.0067 (13)	−0.0136 (14)	0.0017 (13)
C15B	0.060 (2)	0.0466 (19)	0.087 (3)	−0.0192 (16)	−0.0245 (19)	0.0122 (17)
C16B	0.075 (2)	0.055 (2)	0.0545 (19)	−0.0099 (17)	−0.0059 (17)	−0.0169 (16)
C17B	0.0263 (13)	0.0384 (15)	0.0438 (15)	0.0070 (11)	−0.0033 (11)	0.0003 (12)
C18B	0.0368 (16)	0.0461 (16)	0.0445 (16)	0.0100 (13)	−0.0008 (12)	−0.0039 (13)
C19B	0.058 (2)	0.064 (2)	0.0412 (16)	0.0154 (16)	0.0020 (14)	−0.0033 (14)
C20B	0.0512 (19)	0.062 (2)	0.061 (2)	0.0220 (16)	0.0067 (16)	−0.0052 (16)
C21B	0.0314 (13)	0.0238 (12)	0.0250 (12)	0.0027 (10)	0.0000 (10)	0.0026 (9)
C22B	0.0308 (14)	0.0278 (13)	0.0315 (13)	−0.0008 (10)	−0.0017 (11)	0.0031 (10)
C23B	0.0339 (14)	0.0277 (13)	0.0355 (13)	0.0010 (11)	0.0017 (11)	0.0023 (10)
C24B	0.0427 (16)	0.0386 (15)	0.0382 (15)	−0.0105 (12)	−0.0021 (12)	0.0069 (12)
C25B	0.0485 (17)	0.0381 (15)	0.0383 (15)	−0.0106 (13)	0.0028 (13)	0.0057 (12)
C26B	0.066 (2)	0.0454 (17)	0.0491 (17)	−0.0166 (15)	−0.0059 (15)	0.0053 (14)
P1C	0.0314 (4)	0.0241 (3)	0.0302 (3)	0.0007 (3)	0.0001 (3)	0.0031 (2)
O2C	0.0329 (10)	0.0280 (9)	0.0461 (10)	0.0044 (7)	0.0006 (8)	0.0039 (8)
O11C	0.0393 (10)	0.0246 (9)	0.0411 (10)	0.0008 (7)	0.0065 (8)	0.0014 (7)
N12C	0.0343 (12)	0.0254 (10)	0.0318 (11)	0.0037 (9)	−0.0069 (9)	−0.0014 (8)
C3C	0.0305 (13)	0.0253 (12)	0.0309 (13)	−0.0038 (10)	0.0032 (10)	0.0023 (10)
C4C	0.0399 (15)	0.0311 (14)	0.0366 (14)	−0.0015 (12)	0.0048 (12)	−0.0032 (11)
C5C	0.0513 (18)	0.0418 (16)	0.0354 (15)	−0.0061 (14)	0.0059 (13)	−0.0087 (12)
C6C	0.0498 (17)	0.0426 (16)	0.0291 (13)	−0.0153 (13)	−0.0011 (12)	0.0011 (11)
C7C	0.0415 (16)	0.0334 (14)	0.0373 (14)	−0.0040 (12)	−0.0036 (12)	0.0067 (11)
C8C	0.0408 (15)	0.0273 (13)	0.0311 (13)	−0.0022 (11)	0.0018 (11)	0.0023 (10)
C9C	0.0355 (14)	0.0265 (12)	0.0288 (12)	0.0025 (11)	0.0006 (11)	0.0050 (10)
C10C	0.0350 (14)	0.0288 (13)	0.0200 (11)	0.0024 (11)	0.0037 (10)	0.0021 (9)
C13C	0.0402 (15)	0.0248 (13)	0.0355 (14)	0.0032 (11)	−0.0050 (11)	−0.0035 (10)
C14C	0.0403 (15)	0.0360 (14)	0.0358 (14)	−0.0022 (12)	−0.0040 (12)	−0.0037 (11)
C15C	0.0522 (18)	0.0408 (16)	0.0488 (17)	−0.0055 (14)	−0.0060 (14)	−0.0098 (13)
C16C	0.073 (2)	0.0545 (19)	0.0408 (17)	−0.0176 (17)	0.0087 (15)	−0.0133 (14)
C17C	0.0337 (14)	0.0305 (13)	0.0399 (14)	0.0063 (11)	−0.0076 (12)	−0.0012 (11)
C18C	0.0425 (18)	0.0567 (19)	0.061 (2)	0.0146 (15)	0.0013 (15)	0.0061 (15)
C19C	0.0469 (19)	0.091 (3)	0.069 (2)	0.0307 (19)	0.0080 (17)	0.0094 (19)
C20C	0.072 (2)	0.070 (2)	0.0439 (17)	0.0290 (18)	0.0048 (16)	−0.0046 (16)
C21C	0.0350 (14)	0.0297 (13)	0.0294 (13)	0.0005 (11)	−0.0038 (11)	0.0025 (10)
C22C	0.0384 (15)	0.0288 (13)	0.0358 (14)	−0.0016 (11)	0.0005 (11)	0.0016 (11)
C23C	0.0361 (15)	0.0304 (13)	0.0351 (14)	−0.0021 (11)	−0.0018 (11)	−0.0001 (11)
C24C	0.0353 (15)	0.0334 (14)	0.0371 (14)	−0.0023 (11)	0.0000 (11)	−0.0001 (11)
C25C	0.0345 (15)	0.0366 (14)	0.0375 (14)	−0.0035 (12)	−0.0021 (11)	0.0034 (11)
C26C	0.0331 (14)	0.0334 (13)	0.0356 (14)	−0.0047 (11)	−0.0028 (11)	0.0035 (11)
P1D	0.0243 (5)	0.0230 (13)	0.0308 (4)	−0.0019 (7)	−0.0011 (3)	0.0095 (6)
O2D	0.020 (2)	0.014 (3)	0.0468 (16)	−0.009 (2)	−0.0027 (12)	0.009 (2)
O11D	0.0328 (19)	0.0241 (9)	0.026 (8)	−0.0047 (9)	0.009 (3)	0.0022 (17)
N12D	0.0258 (11)	0.0243 (11)	0.0290 (11)	0.0007 (8)	−0.0015 (9)	0.0002 (9)
C3D	0.0290 (14)	0.0292 (14)	0.0323 (17)	−0.0111 (12)	−0.0013 (11)	0.0066 (11)
C4D	0.0290 (14)	0.0292 (14)	0.0323 (17)	−0.0111 (12)	−0.0013 (11)	0.0066 (11)
C5D	0.0290 (14)	0.0292 (14)	0.0323 (17)	−0.0111 (12)	−0.0013 (11)	0.0066 (11)
C6D	0.0290 (14)	0.0292 (14)	0.0323 (17)	−0.0111 (12)	−0.0013 (11)	0.0066 (11)
C7D	0.0290 (14)	0.0292 (14)	0.0323 (17)	−0.0111 (12)	−0.0013 (11)	0.0066 (11)
C8D	0.0290 (14)	0.0292 (14)	0.0323 (17)	−0.0111 (12)	−0.0013 (11)	0.0066 (11)
C9D	0.0276 (13)	0.0273 (12)	0.0245 (14)	−0.0007 (10)	−0.0015 (11)	0.0053 (11)
C10D	0.0274 (13)	0.0266 (13)	0.022 (2)	−0.0015 (10)	0.0020 (11)	0.0046 (11)
C13D	0.0279 (13)	0.0230 (12)	0.032 (2)	−0.0011 (10)	0.0001 (12)	−0.0006 (13)
C14D	0.0422 (16)	0.034 (2)	0.032 (3)	−0.0003 (13)	−0.0023 (15)	−0.0046 (18)
C15D	0.023 (5)	0.0400 (17)	0.046 (3)	0.000 (3)	0.006 (3)	−0.0156 (17)
C16D	0.050 (9)	0.0585 (19)	0.0343 (17)	0.000 (3)	−0.009 (3)	−0.0041 (14)
C17D	0.0290 (14)	0.0266 (14)	0.036 (3)	0.0034 (11)	−0.0049 (14)	0.0033 (17)
C18D	0.0306 (16)	0.023 (2)	0.038 (3)	0.0027 (14)	0.0003 (18)	0.002 (2)
C19D	0.0341 (17)	0.032 (5)	0.054 (7)	0.0067 (17)	0.002 (4)	0.005 (5)
C20D	0.040 (2)	0.032 (5)	0.038 (3)	0.003 (3)	0.001 (2)	−0.002 (3)
C21D	0.019 (2)	0.025 (3)	0.0329 (17)	0.004 (2)	−0.0017 (14)	0.0064 (15)
C22D	0.0321 (18)	0.031 (3)	0.024 (3)	−0.006 (2)	0.001 (2)	−0.0004 (19)
C23D	0.030 (2)	0.031 (2)	0.032 (2)	−0.0058 (17)	−0.0021 (16)	0.0001 (16)
C24D	0.0311 (19)	0.032 (2)	0.033 (2)	−0.0052 (18)	−0.0023 (15)	−0.0023 (16)
C25D	0.034 (3)	0.0340 (19)	0.038 (2)	−0.006 (2)	−0.004 (2)	−0.0004 (16)
C26D	0.039 (4)	0.0332 (18)	0.039 (5)	0.000 (2)	−0.008 (4)	−0.0030 (14)
C27D	0.0447 (17)	0.040 (4)	0.043 (4)	−0.0077 (15)	−0.0010 (14)	−0.006 (3)
C28D	0.062 (8)	0.0411 (18)	0.058 (3)	−0.012 (4)	−0.002 (7)	−0.0152 (16)
P1E	0.0243 (5)	0.0230 (13)	0.0308 (4)	−0.0019 (7)	−0.0011 (3)	0.0095 (6)
O2E	0.020 (2)	0.014 (3)	0.0468 (16)	−0.009 (2)	−0.0027 (12)	0.009 (2)
O11E	0.0328 (19)	0.0241 (9)	0.026 (8)	−0.0047 (9)	0.009 (3)	0.0022 (17)
N12E	0.0258 (11)	0.0243 (11)	0.0290 (11)	0.0007 (8)	−0.0015 (9)	0.0002 (9)
C3E	0.025 (2)	0.035 (2)	0.037 (3)	−0.0100 (17)	0.0054 (16)	0.0070 (17)
C4E	0.025 (2)	0.035 (2)	0.037 (3)	−0.0100 (17)	0.0054 (16)	0.0070 (17)
C5E	0.025 (2)	0.035 (2)	0.037 (3)	−0.0100 (17)	0.0054 (16)	0.0070 (17)
C6E	0.025 (2)	0.035 (2)	0.037 (3)	−0.0100 (17)	0.0054 (16)	0.0070 (17)
C7E	0.025 (2)	0.035 (2)	0.037 (3)	−0.0100 (17)	0.0054 (16)	0.0070 (17)
C8E	0.025 (2)	0.035 (2)	0.037 (3)	−0.0100 (17)	0.0054 (16)	0.0070 (17)
C9E	0.0276 (13)	0.0273 (12)	0.0245 (14)	−0.0007 (10)	−0.0015 (11)	0.0053 (11)
C10E	0.0274 (13)	0.0266 (13)	0.022 (2)	−0.0015 (10)	0.0020 (11)	0.0046 (11)
C13E	0.0279 (13)	0.0230 (12)	0.032 (2)	−0.0011 (10)	0.0001 (12)	−0.0006 (13)
C14E	0.0422 (16)	0.034 (2)	0.032 (3)	−0.0003 (13)	−0.0023 (15)	−0.0046 (18)
C15E	0.023 (5)	0.0400 (17)	0.046 (3)	0.000 (3)	0.006 (3)	−0.0156 (17)
C16E	0.050 (9)	0.0585 (19)	0.0343 (17)	0.000 (3)	−0.009 (3)	−0.0041 (14)
C17E	0.0290 (14)	0.0266 (14)	0.036 (3)	0.0034 (11)	−0.0049 (14)	0.0033 (17)
C18E	0.0306 (16)	0.023 (2)	0.038 (3)	0.0027 (14)	0.0003 (18)	0.002 (2)
C19E	0.0341 (17)	0.032 (5)	0.054 (7)	0.0067 (17)	0.002 (4)	0.005 (5)
C20E	0.040 (2)	0.032 (5)	0.038 (3)	0.003 (3)	0.001 (2)	−0.002 (3)
C21E	0.019 (2)	0.025 (3)	0.0329 (17)	0.004 (2)	−0.0017 (14)	0.0064 (15)
C22E	0.0321 (18)	0.031 (3)	0.024 (3)	−0.006 (2)	0.001 (2)	−0.0004 (19)
C23E	0.030 (2)	0.031 (2)	0.032 (2)	−0.0058 (17)	−0.0021 (16)	0.0001 (16)
C24E	0.0311 (19)	0.032 (2)	0.033 (2)	−0.0052 (18)	−0.0023 (15)	−0.0023 (16)
C25E	0.034 (3)	0.0340 (19)	0.038 (2)	−0.006 (2)	−0.004 (2)	−0.0004 (16)
C26E	0.039 (4)	0.0332 (18)	0.039 (5)	0.000 (2)	−0.008 (4)	−0.0030 (14)
C27E	0.0447 (17)	0.040 (4)	0.043 (4)	−0.0077 (15)	−0.0010 (14)	−0.006 (3)
C28E	0.062 (8)	0.0411 (18)	0.058 (3)	−0.012 (4)	−0.002 (7)	−0.0152 (16)

### Geometric parameters (Å, °)

**Table:**

P1A—O2A	1.4869 (16)	C17C—H17E	0.9900
P1A—C21A	1.798 (2)	C17C—H17F	0.9900
P1A—C3A	1.809 (2)	C18C—C20C	1.474 (4)
P1A—C9A	1.812 (2)	C18C—C19C	1.533 (4)
O11A—C10A	1.233 (3)	C18C—H18C	1.0000
N12A—C10A	1.356 (3)	C19C—H19G	0.9800
N12A—C17A	1.467 (3)	C19C—H19H	0.9800
N12A—C13A	1.471 (3)	C19C—H19I	0.9800
C3A—C8A	1.390 (3)	C20C—H20G	0.9800
C3A—C4A	1.392 (3)	C20C—H20H	0.9800
C4A—C5A	1.389 (3)	C20C—H20I	0.9800
C4A—H4A	0.9500	C21C—C22C	1.525 (3)
C5A—C6A	1.384 (4)	C21C—H21E	0.9900
C5A—H5A	0.9500	C21C—H21F	0.9900
C6A—C7A	1.373 (4)	C22C—C23C	1.520 (3)
C6A—H6A	0.9500	C22C—H22E	0.9900
C7A—C8A	1.386 (4)	C22C—H22F	0.9900
C7A—H7A	0.9500	C23C—C24C	1.523 (3)
C8A—H8A	0.9500	C23C—H23E	0.9900
C9A—C10A	1.517 (3)	C23C—H23F	0.9900
C9A—H9A1	0.9900	C24C—C25C	1.527 (3)
C9A—H9A2	0.9900	C24C—H24E	0.9900
C13A—C14A	1.532 (3)	C24C—H24F	0.9900
C13A—H13A	0.9900	C25C—C26C	1.518 (4)
C13A—H13B	0.9900	C25C—H25E	0.9900
C14A—C15A	1.520 (4)	C25C—H25F	0.9900
C14A—C16A	1.524 (4)	C26C—C27C	1.518 (3)
C14A—H14A	1.0000	C26C—H26E	0.9900
C15A—H15A	0.9800	C26C—H26F	0.9900
C15A—H15B	0.9800	C27C—C28C	1.520 (4)
C15A—H15C	0.9800	C27C—H27E	0.9900
C16A—H16A	0.9800	C27C—H27F	0.9900
C16A—H16B	0.9800	C28C—H28G	0.9800
C16A—H16C	0.9800	C28C—H28H	0.9800
C17A—C18A	1.537 (3)	C28C—H28I	0.9800
C17A—H17A	0.9900	P1D—O2D	1.488 (3)
C17A—H17B	0.9900	P1D—C21D	1.806 (3)
C18A—C20A	1.515 (4)	P1D—C3D	1.812 (2)
C18A—C19A	1.518 (4)	P1D—C9D	1.815 (3)
C18A—H18A	1.0000	O11D—C10D	1.245 (6)
C19A—H19A	0.9800	N12D—C10D	1.349 (4)
C19A—H19B	0.9800	N12D—C13D	1.470 (3)
C19A—H19C	0.9800	N12D—C17D	1.471 (4)
C20A—H20A	0.9800	C3D—C8D	1.3931
C20A—H20B	0.9800	C3D—C4D	1.4030
C20A—H20C	0.9800	C4D—C5D	1.3939
C21A—C22A	1.531 (3)	C4D—H4D	0.9500
C21A—H21A	0.9900	C5D—C6D	1.3924
C21A—H21B	0.9900	C5D—H5D	0.9500
C22A—C23A	1.520 (3)	C6D—C7D	1.4204
C22A—H22A	0.9900	C6D—H6D	0.9500
C22A—H22B	0.9900	C7D—C8D	1.4299
C23A—C24A	1.518 (3)	C7D—H7D	0.9500
C23A—H23A	0.9900	C8D—H8D	0.9500
C23A—H23B	0.9900	C9D—C10D	1.517 (4)
C24A—C25A	1.525 (3)	C9D—H9D1	0.9900
C24A—H24A	0.9900	C9D—H9D2	0.9900
C24A—H24B	0.9900	C13D—C14D	1.532 (4)
C25A—C26A	1.515 (4)	C13D—H13G	0.9900
C25A—H25A	0.9900	C13D—H13H	0.9900
C25A—H25B	0.9900	C14D—C16D	1.518 (5)
C26A—C27A	1.534 (4)	C14D—C15D	1.537 (5)
C26A—H26A	0.9900	C14D—H14D	1.0000
C26A—H26B	0.9900	C15D—H15J	0.9800
C27A—C28A	1.499 (5)	C15D—H15K	0.9800
C27A—H27A	0.9900	C15D—H15L	0.9800
C27A—H27B	0.9900	C16D—H16J	0.9800
C28A—H28A	0.9800	C16D—H16K	0.9800
C28A—H28B	0.9800	C16D—H16L	0.9800
C28A—H28C	0.9800	C17D—C18D	1.524 (4)
P1B—O2B	1.4848 (16)	C17D—H17G	0.9900
P1B—C21B	1.796 (2)	C17D—H17H	0.9900
P1B—C3B	1.812 (2)	C18D—C19D	1.524 (4)
P1B—C9B	1.812 (2)	C18D—C20D	1.525 (4)
O11B—C10B	1.236 (3)	C18D—H18D	1.0000
N12B—C10B	1.356 (3)	C19D—H19J	0.9800
N12B—C13B	1.469 (3)	C19D—H19K	0.9800
N12B—C17B	1.471 (3)	C19D—H19L	0.9800
C3B—C8B	1.388 (3)	C20D—H20J	0.9800
C3B—C4B	1.393 (3)	C20D—H20K	0.9800
C4B—C5B	1.388 (4)	C20D—H20L	0.9800
C4B—H4B	0.9500	C21D—C22D	1.532 (4)
C5B—C6B	1.371 (4)	C21D—H21G	0.9900
C5B—H5B	0.9500	C21D—H21H	0.9900
C6B—C7B	1.387 (4)	C22D—C23D	1.520 (5)
C6B—H6B	0.9500	C22D—H22G	0.9900
C7B—C8B	1.390 (3)	C22D—H22H	0.9900
C7B—H7B	0.9500	C23D—C24D	1.522 (5)
C8B—H8B	0.9500	C23D—H23G	0.9900
C9B—C10B	1.518 (3)	C23D—H23H	0.9900
C9B—H9B1	0.9900	C24D—C25D	1.525 (5)
C9B—H9B2	0.9900	C24D—H24G	0.9900
C13B—C14B	1.511 (4)	C24D—H24H	0.9900
C13B—H13C	0.9900	C25D—C26D	1.513 (5)
C13B—H13D	0.9900	C25D—H25G	0.9900
C14B—C16B	1.486 (4)	C25D—H25H	0.9900
C14B—C15B	1.530 (4)	C26D—C27D	1.523 (6)
C14B—H14B	1.0000	C26D—H26G	0.9900
C15B—H15D	0.9800	C26D—H26H	0.9900
C15B—H15E	0.9800	C27D—C28D	1.520 (5)
C15B—H15F	0.9800	C27D—H27G	0.9900
C16B—H16D	0.9800	C27D—H27H	0.9900
C16B—H16E	0.9800	C28D—H28J	0.9800
C16B—H16F	0.9800	C28D—H28K	0.9800
C17B—C18B	1.532 (4)	C28D—H28L	0.9800
C17B—H17C	0.9900	P1E—O2E	1.488 (3)
C17B—H17D	0.9900	P1E—C21E	1.801 (4)
C18B—C19B	1.516 (4)	P1E—C3E	1.813 (3)
C18B—C20B	1.529 (4)	P1E—C9E	1.820 (4)
C18B—H18B	1.0000	O11E—C10E	1.245 (6)
C19B—H19D	0.9800	N12E—C10E	1.350 (5)
C19B—H19E	0.9800	N12E—C13E	1.470 (4)
C19B—H19F	0.9800	N12E—C17E	1.471 (4)
C20B—H20D	0.9800	C3E—C8E	1.3951
C20B—H20E	0.9800	C3E—C4E	1.4033
C20B—H20F	0.9800	C4E—C5E	1.3983
C21B—C22B	1.531 (3)	C4E—H4E	0.9500
C21B—H21C	0.9900	C5E—C6E	1.3958
C21B—H21D	0.9900	C5E—H5E	0.9500
C22B—C23B	1.525 (3)	C6E—C7E	1.4237
C22B—H22C	0.9900	C6E—H6E	0.9500
C22B—H22D	0.9900	C7E—C8E	1.4350
C23B—C24B	1.515 (3)	C7E—H7E	0.9500
C23B—H23C	0.9900	C8E—H8E	0.9500
C23B—H23D	0.9900	C9E—C10E	1.517 (4)
C24B—C25B	1.533 (4)	C9E—H9E1	0.9900
C24B—H24C	0.9900	C9E—H9E2	0.9900
C24B—H24D	0.9900	C13E—C14E	1.532 (5)
C25B—C26B	1.511 (4)	C13E—H13I	0.9900
C25B—H25C	0.9900	C13E—H13J	0.9900
C25B—H25D	0.9900	C14E—C16E	1.518 (5)
C26B—C27B	1.528 (4)	C14E—C15E	1.538 (5)
C26B—H26C	0.9900	C14E—H14E	1.0000
C26B—H26D	0.9900	C15E—H15M	0.9800
C27B—C28B	1.516 (4)	C15E—H15N	0.9800
C27B—H27C	0.9900	C15E—H15O	0.9800
C27B—H27D	0.9900	C16E—H16M	0.9800
C28B—H28D	0.9800	C16E—H16N	0.9800
C28B—H28E	0.9800	C16E—H16O	0.9800
C28B—H28F	0.9800	C17E—C18E	1.524 (5)
P1C—O2C	1.4873 (17)	C17E—H17I	0.9900
P1C—C21C	1.800 (2)	C17E—H17J	0.9900
P1C—C9C	1.812 (2)	C18E—C19E	1.524 (5)
P1C—C3C	1.812 (2)	C18E—C20E	1.525 (5)
O11C—C10C	1.240 (3)	C18E—H18E	1.0000
N12C—C10C	1.347 (3)	C19E—H19M	0.9800
N12C—C17C	1.465 (3)	C19E—H19N	0.9800
N12C—C13C	1.471 (3)	C19E—H19O	0.9800
C3C—C4C	1.392 (3)	C20E—H20M	0.9800
C3C—C8C	1.396 (3)	C20E—H20N	0.9800
C4C—C5C	1.389 (4)	C20E—H20O	0.9800
C4C—H4C	0.9500	C21E—C22E	1.525 (5)
C5C—C6C	1.385 (4)	C21E—H21I	0.9900
C5C—H5C	0.9500	C21E—H21J	0.9900
C6C—C7C	1.391 (4)	C22E—C23E	1.522 (6)
C6C—H6C	0.9500	C22E—H22I	0.9900
C7C—C8C	1.388 (3)	C22E—H22J	0.9900
C7C—H7C	0.9500	C23E—C24E	1.521 (5)
C8C—H8C	0.9500	C23E—H23I	0.9900
C9C—C10C	1.517 (3)	C23E—H23J	0.9900
C9C—H9C1	0.9900	C24E—C25E	1.521 (6)
C9C—H9C2	0.9900	C24E—H24I	0.9900
C13C—C14C	1.523 (3)	C24E—H24J	0.9900
C13C—H13E	0.9900	C25E—C26E	1.515 (6)
C13C—H13F	0.9900	C25E—H25I	0.9900
C14C—C16C	1.500 (4)	C25E—H25J	0.9900
C14C—C15C	1.522 (4)	C26E—C27E	1.522 (6)
C14C—H14C	1.0000	C26E—H26I	0.9900
C15C—H15G	0.9800	C26E—H26J	0.9900
C15C—H15H	0.9800	C27E—C28E	1.520 (6)
C15C—H15I	0.9800	C27E—H27I	0.9900
C16C—H16G	0.9800	C27E—H27J	0.9900
C16C—H16H	0.9800	C28E—H28M	0.9800
C16C—H16I	0.9800	C28E—H28N	0.9800
C17C—C18C	1.498 (4)	C28E—H28O	0.9800
			
O2A—P1A—C21A	112.52 (10)	C23C—C22C—C21C	113.4 (2)
O2A—P1A—C3A	111.92 (10)	C23C—C22C—H22E	108.9
C21A—P1A—C3A	106.61 (11)	C21C—C22C—H22E	108.9
O2A—P1A—C9A	110.57 (10)	C23C—C22C—H22F	108.9
C21A—P1A—C9A	108.17 (11)	C21C—C22C—H22F	108.9
C3A—P1A—C9A	106.78 (11)	H22E—C22C—H22F	107.7
C10A—N12A—C17A	124.72 (19)	C22C—C23C—C24C	113.8 (2)
C10A—N12A—C13A	117.00 (19)	C22C—C23C—H23E	108.8
C17A—N12A—C13A	118.20 (19)	C24C—C23C—H23E	108.8
C8A—C3A—C4A	118.7 (2)	C22C—C23C—H23F	108.8
C8A—C3A—P1A	118.37 (18)	C24C—C23C—H23F	108.8
C4A—C3A—P1A	122.90 (18)	H23E—C23C—H23F	107.7
C5A—C4A—C3A	120.9 (2)	C23C—C24C—C25C	114.2 (2)
C5A—C4A—H4A	119.6	C23C—C24C—H24E	108.7
C3A—C4A—H4A	119.6	C25C—C24C—H24E	108.7
C6A—C5A—C4A	119.3 (2)	C23C—C24C—H24F	108.7
C6A—C5A—H5A	120.3	C25C—C24C—H24F	108.7
C4A—C5A—H5A	120.3	H24E—C24C—H24F	107.6
C7A—C6A—C5A	120.5 (2)	C26C—C25C—C24C	114.7 (2)
C7A—C6A—H6A	119.8	C26C—C25C—H25E	108.6
C5A—C6A—H6A	119.8	C24C—C25C—H25E	108.6
C6A—C7A—C8A	120.2 (3)	C26C—C25C—H25F	108.6
C6A—C7A—H7A	119.9	C24C—C25C—H25F	108.6
C8A—C7A—H7A	119.9	H25E—C25C—H25F	107.6
C7A—C8A—C3A	120.4 (2)	C27C—C26C—C25C	113.4 (2)
C7A—C8A—H8A	119.8	C27C—C26C—H26E	108.9
C3A—C8A—H8A	119.8	C25C—C26C—H26E	108.9
C10A—C9A—P1A	116.92 (16)	C27C—C26C—H26F	108.9
C10A—C9A—H9A1	108.1	C25C—C26C—H26F	108.9
P1A—C9A—H9A1	108.1	H26E—C26C—H26F	107.7
C10A—C9A—H9A2	108.1	C26C—C27C—C28C	113.3 (2)
P1A—C9A—H9A2	108.1	C26C—C27C—H27E	108.9
H9A1—C9A—H9A2	107.3	C28C—C27C—H27E	108.9
O11A—C10A—N12A	121.2 (2)	C26C—C27C—H27F	108.9
O11A—C10A—C9A	120.2 (2)	C28C—C27C—H27F	108.9
N12A—C10A—C9A	118.6 (2)	H27E—C27C—H27F	107.7
N12A—C13A—C14A	114.9 (2)	O2D—P1D—C21D	112.3 (2)
N12A—C13A—H13A	108.5	O2D—P1D—C3D	111.6 (2)
C14A—C13A—H13A	108.5	C21D—P1D—C3D	106.0 (2)
N12A—C13A—H13B	108.5	O2D—P1D—C9D	110.9 (3)
C14A—C13A—H13B	108.5	C21D—P1D—C9D	108.0 (3)
H13A—C13A—H13B	107.5	C3D—P1D—C9D	107.8 (2)
C15A—C14A—C16A	110.9 (2)	C10D—N12D—C13D	124.7 (4)
C15A—C14A—C13A	112.2 (2)	C10D—N12D—C17D	118.1 (4)
C16A—C14A—C13A	108.3 (2)	C13D—N12D—C17D	117.1 (3)
C15A—C14A—H14A	108.5	C8D—C3D—C4D	120.2
C16A—C14A—H14A	108.5	C8D—C3D—P1D	117.2 (2)
C13A—C14A—H14A	108.5	C4D—C3D—P1D	122.5 (2)
N12A—C17A—C18A	114.8 (2)	C5D—C4D—C3D	119.9
N12A—C17A—H17A	108.6	C5D—C4D—H4D	120.1
C18A—C17A—H17A	108.6	C3D—C4D—H4D	120.1
N12A—C17A—H17B	108.6	C6D—C5D—C4D	120.5
C18A—C17A—H17B	108.6	C6D—C5D—H5D	119.8
H17A—C17A—H17B	107.5	C4D—C5D—H5D	119.8
C20A—C18A—C19A	111.5 (2)	C5D—C6D—C7D	119.5
C20A—C18A—C17A	113.4 (2)	C5D—C6D—H6D	120.2
C19A—C18A—C17A	108.1 (2)	C7D—C6D—H6D	120.2
C20A—C18A—H18A	107.9	C6D—C7D—C8D	117.4
C19A—C18A—H18A	107.9	C6D—C7D—H7D	121.3
C17A—C18A—H18A	107.9	C8D—C7D—H7D	121.3
C22A—C21A—P1A	112.98 (16)	C3D—C8D—C7D	119.3
C22A—C21A—H21A	109.0	C3D—C8D—H8D	120.3
P1A—C21A—H21A	109.0	C7D—C8D—H8D	120.3
C22A—C21A—H21B	109.0	C10D—C9D—P1D	116.2 (4)
P1A—C21A—H21B	109.0	C10D—C9D—H9D1	108.2
H21A—C21A—H21B	107.8	P1D—C9D—H9D1	108.2
C23A—C22A—C21A	111.67 (19)	C10D—C9D—H9D2	108.2
C23A—C22A—H22A	109.3	P1D—C9D—H9D2	108.2
C21A—C22A—H22A	109.3	H9D1—C9D—H9D2	107.4
C23A—C22A—H22B	109.3	O11D—C10D—N12D	121.0 (5)
C21A—C22A—H22B	109.3	O11D—C10D—C9D	118.7 (5)
H22A—C22A—H22B	107.9	N12D—C10D—C9D	118.9 (4)
C24A—C23A—C22A	114.1 (2)	N12D—C13D—C14D	114.6 (4)
C24A—C23A—H23A	108.7	N12D—C13D—H13G	108.6
C22A—C23A—H23A	108.7	C14D—C13D—H13G	108.6
C24A—C23A—H23B	108.7	N12D—C13D—H13H	108.6
C22A—C23A—H23B	108.7	C14D—C13D—H13H	108.6
H23A—C23A—H23B	107.6	H13G—C13D—H13H	107.6
C23A—C24A—C25A	112.5 (2)	C16D—C14D—C13D	113.6 (4)
C23A—C24A—H24A	109.1	C16D—C14D—C15D	112.4 (4)
C25A—C24A—H24A	109.1	C13D—C14D—C15D	108.0 (4)
C23A—C24A—H24B	109.1	C16D—C14D—H14D	107.5
C25A—C24A—H24B	109.1	C13D—C14D—H14D	107.5
H24A—C24A—H24B	107.8	C15D—C14D—H14D	107.5
C26A—C25A—C24A	114.2 (2)	N12D—C17D—C18D	114.1 (4)
C26A—C25A—H25A	108.7	N12D—C17D—H17G	108.7
C24A—C25A—H25A	108.7	C18D—C17D—H17G	108.7
C26A—C25A—H25B	108.7	N12D—C17D—H17H	108.7
C24A—C25A—H25B	108.7	C18D—C17D—H17H	108.7
H25A—C25A—H25B	107.6	H17G—C17D—H17H	107.6
C25A—C26A—C27A	113.1 (2)	C19D—C18D—C17D	108.0 (4)
C25A—C26A—H26A	109.0	C19D—C18D—C20D	111.6 (4)
C27A—C26A—H26A	109.0	C17D—C18D—C20D	113.0 (4)
C25A—C26A—H26B	109.0	C19D—C18D—H18D	108.0
C27A—C26A—H26B	109.0	C17D—C18D—H18D	108.0
H26A—C26A—H26B	107.8	C20D—C18D—H18D	108.0
C28A—C27A—C26A	114.2 (3)	C22D—C21D—P1D	112.0 (3)
C28A—C27A—H27A	108.7	C22D—C21D—H21G	109.2
C26A—C27A—H27A	108.7	P1D—C21D—H21G	109.2
C28A—C27A—H27B	108.7	C22D—C21D—H21H	109.2
C26A—C27A—H27B	108.7	P1D—C21D—H21H	109.2
H27A—C27A—H27B	107.6	H21G—C21D—H21H	107.9
O2B—P1B—C21B	113.30 (10)	C23D—C22D—C21D	112.8 (4)
O2B—P1B—C3B	111.95 (10)	C23D—C22D—H22G	109.0
C21B—P1B—C3B	106.33 (11)	C21D—C22D—H22G	109.0
O2B—P1B—C9B	110.78 (10)	C23D—C22D—H22H	109.0
C21B—P1B—C9B	106.80 (11)	C21D—C22D—H22H	109.0
C3B—P1B—C9B	107.31 (11)	H22G—C22D—H22H	107.8
C10B—N12B—C13B	125.6 (2)	C22D—C23D—C24D	115.2 (3)
C10B—N12B—C17B	117.3 (2)	C22D—C23D—H23G	108.5
C13B—N12B—C17B	116.6 (2)	C24D—C23D—H23G	108.5
C8B—C3B—C4B	119.2 (2)	C22D—C23D—H23H	108.5
C8B—C3B—P1B	122.73 (18)	C24D—C23D—H23H	108.5
C4B—C3B—P1B	118.06 (18)	H23G—C23D—H23H	107.5
C5B—C4B—C3B	120.0 (2)	C23D—C24D—C25D	111.7 (3)
C5B—C4B—H4B	120.0	C23D—C24D—H24G	109.3
C3B—C4B—H4B	120.0	C25D—C24D—H24G	109.3
C6B—C5B—C4B	120.3 (2)	C23D—C24D—H24H	109.3
C6B—C5B—H5B	119.8	C25D—C24D—H24H	109.3
C4B—C5B—H5B	119.8	H24G—C24D—H24H	107.9
C5B—C6B—C7B	120.4 (2)	C26D—C25D—C24D	115.8 (4)
C5B—C6B—H6B	119.8	C26D—C25D—H25G	108.3
C7B—C6B—H6B	119.8	C24D—C25D—H25G	108.3
C6B—C7B—C8B	119.4 (2)	C26D—C25D—H25H	108.3
C6B—C7B—H7B	120.3	C24D—C25D—H25H	108.3
C8B—C7B—H7B	120.3	H25G—C25D—H25H	107.4
C3B—C8B—C7B	120.6 (2)	C25D—C26D—C27D	113.3 (4)
C3B—C8B—H8B	119.7	C25D—C26D—H26G	108.9
C7B—C8B—H8B	119.7	C27D—C26D—H26G	108.9
C10B—C9B—P1B	116.17 (16)	C25D—C26D—H26H	108.9
C10B—C9B—H9B1	108.2	C27D—C26D—H26H	108.9
P1B—C9B—H9B1	108.2	H26G—C26D—H26H	107.7
C10B—C9B—H9B2	108.2	C28D—C27D—C26D	113.6 (5)
P1B—C9B—H9B2	108.2	C28D—C27D—H27G	108.8
H9B1—C9B—H9B2	107.4	C26D—C27D—H27G	108.8
O11B—C10B—N12B	120.8 (2)	C28D—C27D—H27H	108.8
O11B—C10B—C9B	119.4 (2)	C26D—C27D—H27H	108.8
N12B—C10B—C9B	119.8 (2)	H27G—C27D—H27H	107.7
N12B—C13B—C14B	113.9 (2)	O2E—P1E—C21E	113.2 (4)
N12B—C13B—H13C	108.8	O2E—P1E—C3E	111.3 (4)
C14B—C13B—H13C	108.8	C21E—P1E—C3E	107.3 (3)
N12B—C13B—H13D	108.8	O2E—P1E—C9E	110.1 (4)
C14B—C13B—H13D	108.8	C21E—P1E—C9E	107.6 (3)
H13C—C13B—H13D	107.7	C3E—P1E—C9E	107.1 (3)
C16B—C14B—C13B	112.8 (2)	C10E—N12E—C13E	124.4 (6)
C16B—C14B—C15B	110.2 (3)	C10E—N12E—C17E	117.8 (5)
C13B—C14B—C15B	108.3 (2)	C13E—N12E—C17E	117.0 (5)
C16B—C14B—H14B	108.5	C8E—C3E—C4E	119.9
C13B—C14B—H14B	108.5	C8E—C3E—P1E	117.8 (3)
C15B—C14B—H14B	108.5	C4E—C3E—P1E	122.3 (3)
N12B—C17B—C18B	115.2 (2)	C5E—C4E—C3E	120.1
N12B—C17B—H17C	108.5	C5E—C4E—H4E	119.9
C18B—C17B—H17C	108.5	C3E—C4E—H4E	119.9
N12B—C17B—H17D	108.5	C6E—C5E—C4E	119.8
C18B—C17B—H17D	108.5	C6E—C5E—H5E	120.1
H17C—C17B—H17D	107.5	C4E—C5E—H5E	120.1
C19B—C18B—C20B	110.7 (2)	C5E—C6E—C7E	119.2
C19B—C18B—C17B	112.6 (2)	C5E—C6E—H6E	120.4
C20B—C18B—C17B	108.0 (2)	C7E—C6E—H6E	120.4
C19B—C18B—H18B	108.5	C6E—C7E—C8E	116.1
C20B—C18B—H18B	108.5	C6E—C7E—H7E	122.0
C17B—C18B—H18B	108.5	C8E—C7E—H7E	122.0
C22B—C21B—P1B	113.78 (16)	C3E—C8E—C7E	118.5
C22B—C21B—H21C	108.8	C3E—C8E—H8E	120.8
P1B—C21B—H21C	108.8	C7E—C8E—H8E	120.8
C22B—C21B—H21D	108.8	C10E—C9E—P1E	115.9 (4)
P1B—C21B—H21D	108.8	C10E—C9E—H9E1	108.3
H21C—C21B—H21D	107.7	P1E—C9E—H9E1	108.3
C23B—C22B—C21B	111.00 (19)	C10E—C9E—H9E2	108.3
C23B—C22B—H22C	109.4	P1E—C9E—H9E2	108.3
C21B—C22B—H22C	109.4	H9E1—C9E—H9E2	107.4
C23B—C22B—H22D	109.4	O11E—C10E—N12E	120.7 (8)
C21B—C22B—H22D	109.4	O11E—C10E—C9E	118.6 (6)
H22C—C22B—H22D	108.0	N12E—C10E—C9E	118.9 (6)
C24B—C23B—C22B	114.2 (2)	N12E—C13E—C14E	114.7 (5)
C24B—C23B—H23C	108.7	N12E—C13E—H13I	108.6
C22B—C23B—H23C	108.7	C14E—C13E—H13I	108.6
C24B—C23B—H23D	108.7	N12E—C13E—H13J	108.6
C22B—C23B—H23D	108.7	C14E—C13E—H13J	108.6
H23C—C23B—H23D	107.6	H13I—C13E—H13J	107.6
C23B—C24B—C25B	112.1 (2)	C16E—C14E—C13E	113.6 (5)
C23B—C24B—H24C	109.2	C16E—C14E—C15E	112.3 (5)
C25B—C24B—H24C	109.2	C13E—C14E—C15E	107.9 (5)
C23B—C24B—H24D	109.2	C16E—C14E—H14E	107.6
C25B—C24B—H24D	109.2	C13E—C14E—H14E	107.6
H24C—C24B—H24D	107.9	C15E—C14E—H14E	107.6
C26B—C25B—C24B	114.2 (2)	C14E—C15E—H15M	109.5
C26B—C25B—H25C	108.7	C14E—C15E—H15N	109.5
C24B—C25B—H25C	108.7	H15M—C15E—H15N	109.5
C26B—C25B—H25D	108.7	C14E—C15E—H15O	109.5
C24B—C25B—H25D	108.7	H15M—C15E—H15O	109.5
H25C—C25B—H25D	107.6	H15N—C15E—H15O	109.5
C25B—C26B—C27B	113.9 (3)	C14E—C16E—H16M	109.5
C25B—C26B—H26C	108.8	C14E—C16E—H16N	109.5
C27B—C26B—H26C	108.8	H16M—C16E—H16N	109.5
C25B—C26B—H26D	108.8	C14E—C16E—H16O	109.5
C27B—C26B—H26D	108.8	H16M—C16E—H16O	109.5
H26C—C26B—H26D	107.7	H16N—C16E—H16O	109.5
C28B—C27B—C26B	114.5 (3)	N12E—C17E—C18E	114.1 (5)
C28B—C27B—H27C	108.6	N12E—C17E—H17I	108.7
C26B—C27B—H27C	108.6	C18E—C17E—H17I	108.7
C28B—C27B—H27D	108.6	N12E—C17E—H17J	108.7
C26B—C27B—H27D	108.6	C18E—C17E—H17J	108.7
H27C—C27B—H27D	107.6	H17I—C17E—H17J	107.6
O2C—P1C—C21C	112.86 (11)	C19E—C18E—C17E	108.1 (5)
O2C—P1C—C9C	109.98 (10)	C19E—C18E—C20E	111.5 (5)
C21C—P1C—C9C	108.27 (11)	C17E—C18E—C20E	113.0 (5)
O2C—P1C—C3C	111.90 (11)	C19E—C18E—H18E	108.0
C21C—P1C—C3C	107.04 (11)	C17E—C18E—H18E	108.0
C9C—P1C—C3C	106.52 (11)	C20E—C18E—H18E	108.0
C10C—N12C—C17C	117.6 (2)	C18E—C19E—H19M	109.5
C10C—N12C—C13C	126.0 (2)	C18E—C19E—H19N	109.5
C17C—N12C—C13C	116.3 (2)	H19M—C19E—H19N	109.5
C4C—C3C—C8C	119.2 (2)	C18E—C19E—H19O	109.5
C4C—C3C—P1C	117.82 (19)	H19M—C19E—H19O	109.5
C8C—C3C—P1C	122.99 (18)	H19N—C19E—H19O	109.5
C5C—C4C—C3C	120.1 (2)	C18E—C20E—H20M	109.5
C5C—C4C—H4C	120.0	C18E—C20E—H20N	109.5
C3C—C4C—H4C	120.0	H20M—C20E—H20N	109.5
C6C—C5C—C4C	120.5 (2)	C18E—C20E—H20O	109.5
C6C—C5C—H5C	119.7	H20M—C20E—H20O	109.5
C4C—C5C—H5C	119.7	H20N—C20E—H20O	109.5
C5C—C6C—C7C	119.8 (2)	C22E—C21E—P1E	114.2 (4)
C5C—C6C—H6C	120.1	C22E—C21E—H21I	108.7
C7C—C6C—H6C	120.1	P1E—C21E—H21I	108.7
C8C—C7C—C6C	119.8 (2)	C22E—C21E—H21J	108.7
C8C—C7C—H7C	120.1	P1E—C21E—H21J	108.7
C6C—C7C—H7C	120.1	H21I—C21E—H21J	107.6
C7C—C8C—C3C	120.7 (2)	C23E—C22E—C21E	113.8 (5)
C7C—C8C—H8C	119.7	C23E—C22E—H22I	108.8
C3C—C8C—H8C	119.7	C21E—C22E—H22I	108.8
C10C—C9C—P1C	116.54 (16)	C23E—C22E—H22J	108.8
C10C—C9C—H9C1	108.2	C21E—C22E—H22J	108.8
P1C—C9C—H9C1	108.2	H22I—C22E—H22J	107.7
C10C—C9C—H9C2	108.2	C24E—C23E—C22E	114.0 (5)
P1C—C9C—H9C2	108.2	C24E—C23E—H23I	108.7
H9C1—C9C—H9C2	107.3	C22E—C23E—H23I	108.7
O11C—C10C—N12C	121.2 (2)	C24E—C23E—H23J	108.7
O11C—C10C—C9C	118.5 (2)	C22E—C23E—H23J	108.7
N12C—C10C—C9C	120.2 (2)	H23I—C23E—H23J	107.6
N12C—C13C—C14C	113.7 (2)	C25E—C24E—C23E	114.5 (4)
N12C—C13C—H13E	108.8	C25E—C24E—H24I	108.6
C14C—C13C—H13E	108.8	C23E—C24E—H24I	108.6
N12C—C13C—H13F	108.8	C25E—C24E—H24J	108.6
C14C—C13C—H13F	108.8	C23E—C24E—H24J	108.6
H13E—C13C—H13F	107.7	H24I—C24E—H24J	107.6
C16C—C14C—C15C	111.4 (2)	C26E—C25E—C24E	115.6 (5)
C16C—C14C—C13C	112.3 (2)	C26E—C25E—H25I	108.4
C15C—C14C—C13C	109.1 (2)	C24E—C25E—H25I	108.4
C16C—C14C—H14C	107.9	C26E—C25E—H25J	108.4
C15C—C14C—H14C	107.9	C24E—C25E—H25J	108.4
C13C—C14C—H14C	107.9	H25I—C25E—H25J	107.4
N12C—C17C—C18C	113.7 (2)	C25E—C26E—C27E	113.4 (5)
N12C—C17C—H17E	108.8	C25E—C26E—H26I	108.9
C18C—C17C—H17E	108.8	C27E—C26E—H26I	108.9
N12C—C17C—H17F	108.8	C25E—C26E—H26J	108.9
C18C—C17C—H17F	108.8	C27E—C26E—H26J	108.9
H17E—C17C—H17F	107.7	H26I—C26E—H26J	107.7
C20C—C18C—C17C	114.7 (3)	C28E—C27E—C26E	113.5 (6)
C20C—C18C—C19C	111.6 (3)	C28E—C27E—H27I	108.9
C17C—C18C—C19C	107.7 (2)	C26E—C27E—H27I	108.9
C20C—C18C—H18C	107.5	C28E—C27E—H27J	108.9
C17C—C18C—H18C	107.5	C26E—C27E—H27J	108.9
C19C—C18C—H18C	107.5	H27I—C27E—H27J	107.7
C22C—C21C—P1C	113.37 (17)	C27E—C28E—H28M	109.5
C22C—C21C—H21E	108.9	C27E—C28E—H28N	109.5
P1C—C21C—H21E	108.9	H28M—C28E—H28N	109.5
C22C—C21C—H21F	108.9	C27E—C28E—H28O	109.5
P1C—C21C—H21F	108.9	H28M—C28E—H28O	109.5
H21E—C21C—H21F	107.7	H28N—C28E—H28O	109.5
			
O2A—P1A—C3A—C8A	−11.3 (2)	C13C—N12C—C10C—C9C	−1.0 (3)
C21A—P1A—C3A—C8A	−134.72 (19)	P1C—C9C—C10C—O11C	46.2 (3)
C9A—P1A—C3A—C8A	109.8 (2)	P1C—C9C—C10C—N12C	−135.55 (19)
O2A—P1A—C3A—C4A	169.38 (19)	C10C—N12C—C13C—C14C	121.0 (3)
C21A—P1A—C3A—C4A	46.0 (2)	C17C—N12C—C13C—C14C	−63.4 (3)
C9A—P1A—C3A—C4A	−69.5 (2)	N12C—C13C—C14C—C16C	−51.6 (3)
C8A—C3A—C4A—C5A	−0.1 (4)	N12C—C13C—C14C—C15C	−175.7 (2)
P1A—C3A—C4A—C5A	179.2 (2)	C10C—N12C—C17C—C18C	102.0 (3)
C3A—C4A—C5A—C6A	0.3 (4)	C13C—N12C—C17C—C18C	−74.1 (3)
C4A—C5A—C6A—C7A	−0.5 (4)	N12C—C17C—C18C—C20C	−53.5 (3)
C5A—C6A—C7A—C8A	0.5 (4)	N12C—C17C—C18C—C19C	−178.4 (3)
C6A—C7A—C8A—C3A	−0.4 (4)	O2C—P1C—C21C—C22C	−61.5 (2)
C4A—C3A—C8A—C7A	0.2 (4)	C9C—P1C—C21C—C22C	176.53 (17)
P1A—C3A—C8A—C7A	−179.2 (2)	C3C—P1C—C21C—C22C	62.1 (2)
O2A—P1A—C9A—C10A	175.96 (16)	P1C—C21C—C22C—C23C	173.37 (17)
C21A—P1A—C9A—C10A	−60.4 (2)	C21C—C22C—C23C—C24C	177.2 (2)
C3A—P1A—C9A—C10A	53.98 (19)	C22C—C23C—C24C—C25C	166.3 (2)
C17A—N12A—C10A—O11A	177.9 (2)	C23C—C24C—C25C—C26C	60.0 (3)
C13A—N12A—C10A—O11A	1.2 (3)	C24C—C25C—C26C—C27C	177.0 (2)
C17A—N12A—C10A—C9A	0.1 (3)	C25C—C26C—C27C—C28C	175.7 (2)
C13A—N12A—C10A—C9A	−176.58 (19)	O2D—P1D—C3D—C8D	0.1 (4)
P1A—C9A—C10A—O11A	52.0 (3)	C21D—P1D—C3D—C8D	−122.5 (3)
P1A—C9A—C10A—N12A	−130.17 (19)	C9D—P1D—C3D—C8D	122.0 (3)
C10A—N12A—C13A—C14A	−79.2 (3)	O2D—P1D—C3D—C4D	178.6 (3)
C17A—N12A—C13A—C14A	103.9 (2)	C21D—P1D—C3D—C4D	56.0 (3)
N12A—C13A—C14A—C15A	−52.6 (3)	C9D—P1D—C3D—C4D	−59.4 (4)
N12A—C13A—C14A—C16A	−175.3 (2)	C8D—C3D—C4D—C5D	−1.2
C10A—N12A—C17A—C18A	−80.3 (3)	P1D—C3D—C4D—C5D	−179.7 (3)
C13A—N12A—C17A—C18A	96.4 (2)	C3D—C4D—C5D—C6D	0.8
N12A—C17A—C18A—C20A	−53.8 (3)	C4D—C5D—C6D—C7D	10.2
N12A—C17A—C18A—C19A	−177.9 (2)	C5D—C6D—C7D—C8D	−20.4
O2A—P1A—C21A—C22A	−57.23 (19)	C4D—C3D—C8D—C7D	−9.3
C3A—P1A—C21A—C22A	65.82 (19)	P1D—C3D—C8D—C7D	169.2 (3)
C9A—P1A—C21A—C22A	−179.66 (16)	C6D—C7D—C8D—C3D	19.9
P1A—C21A—C22A—C23A	−171.83 (17)	O2D—P1D—C9D—C10D	170.9 (9)
C21A—C22A—C23A—C24A	179.8 (2)	C21D—P1D—C9D—C10D	−65.6 (9)
C22A—C23A—C24A—C25A	−167.6 (2)	C3D—P1D—C9D—C10D	48.5 (9)
C23A—C24A—C25A—C26A	170.4 (2)	C13D—N12D—C10D—O11D	174.7 (17)
C24A—C25A—C26A—C27A	172.8 (2)	C17D—N12D—C10D—O11D	−10 (3)
C25A—C26A—C27A—C28A	52.3 (4)	C13D—N12D—C10D—C9D	9(3)
O2B—P1B—C3B—C8B	171.00 (19)	C17D—N12D—C10D—C9D	−176.1 (11)
C21B—P1B—C3B—C8B	46.8 (2)	P1D—C9D—C10D—O11D	60 (2)
C9B—P1B—C3B—C8B	−67.2 (2)	P1D—C9D—C10D—N12D	−133.3 (19)
O2B—P1B—C3B—C4B	−7.4 (2)	C10D—N12D—C13D—C14D	81 (2)
C21B—P1B—C3B—C4B	−131.59 (19)	C17D—N12D—C13D—C14D	−94.0 (14)
C9B—P1B—C3B—C4B	114.40 (19)	N12D—C13D—C14D—C16D	56.4 (10)
C8B—C3B—C4B—C5B	0.0 (4)	N12D—C13D—C14D—C15D	−178.2 (9)
P1B—C3B—C4B—C5B	178.5 (2)	C10D—N12D—C17D—C18D	96.0 (17)
C3B—C4B—C5B—C6B	0.4 (4)	C13D—N12D—C17D—C18D	−88.2 (17)
C4B—C5B—C6B—C7B	−0.6 (4)	N12D—C17D—C18D—C19D	177.8 (13)
C5B—C6B—C7B—C8B	0.4 (4)	N12D—C17D—C18D—C20D	−58.2 (13)
C4B—C3B—C8B—C7B	−0.2 (4)	O2D—P1D—C21D—C22D	−67.3 (6)
P1B—C3B—C8B—C7B	−178.55 (19)	C3D—P1D—C21D—C22D	54.8 (5)
C6B—C7B—C8B—C3B	0.0 (4)	C9D—P1D—C21D—C22D	170.1 (5)
O2B—P1B—C9B—C10B	171.94 (16)	P1D—C21D—C22D—C23D	156.4 (4)
C21B—P1B—C9B—C10B	−64.2 (2)	C21D—C22D—C23D—C24D	68.3 (6)
C3B—P1B—C9B—C10B	49.5 (2)	C22D—C23D—C24D—C25D	169.8 (4)
C13B—N12B—C10B—O11B	176.6 (2)	C23D—C24D—C25D—C26D	177.4 (7)
C17B—N12B—C10B—O11B	5.0 (3)	C24D—C25D—C26D—C27D	177 (2)
C13B—N12B—C10B—C9B	−2.8 (3)	C25D—C26D—C27D—C28D	172 (3)
C17B—N12B—C10B—C9B	−174.5 (2)	O2E—P1E—C3E—C8E	−6.8 (5)
P1B—C9B—C10B—O11B	59.8 (3)	C21E—P1E—C3E—C8E	−131.1 (4)
P1B—C9B—C10B—N12B	−120.7 (2)	C9E—P1E—C3E—C8E	113.6 (5)
C10B—N12B—C13B—C14B	−98.6 (3)	O2E—P1E—C3E—C4E	172.8 (5)
C17B—N12B—C13B—C14B	73.2 (3)	C21E—P1E—C3E—C4E	48.4 (5)
N12B—C13B—C14B—C16B	58.8 (3)	C9E—P1E—C3E—C4E	−66.8 (5)
N12B—C13B—C14B—C15B	−179.0 (2)	C8E—C3E—C4E—C5E	1.6
C10B—N12B—C17B—C18B	−83.4 (3)	P1E—C3E—C4E—C5E	−178.0 (5)
C13B—N12B—C17B—C18B	104.1 (3)	C3E—C4E—C5E—C6E	−3.1
N12B—C17B—C18B—C19B	−56.4 (3)	C4E—C5E—C6E—C7E	−12.0
N12B—C17B—C18B—C20B	−179.0 (2)	C5E—C6E—C7E—C8E	27.6
O2B—P1B—C21B—C22B	−61.62 (19)	C4E—C3E—C8E—C7E	14.7
C3B—P1B—C21B—C22B	61.78 (19)	P1E—C3E—C8E—C7E	−165.7 (4)
C9B—P1B—C21B—C22B	176.13 (17)	C6E—C7E—C8E—C3E	−28.9
P1B—C21B—C22B—C23B	179.78 (17)	O2E—P1E—C9E—C10E	−176.0 (13)
C21B—C22B—C23B—C24B	176.8 (2)	C21E—P1E—C9E—C10E	−52.2 (14)
C22B—C23B—C24B—C25B	−172.3 (2)	C3E—P1E—C9E—C10E	62.9 (14)
C23B—C24B—C25B—C26B	165.0 (3)	C13E—N12E—C10E—O11E	−173 (3)
C24B—C25B—C26B—C27B	−179.1 (3)	C17E—N12E—C10E—O11E	17 (5)
C25B—C26B—C27B—C28B	58.7 (4)	C13E—N12E—C10E—C9E	−9(5)
O2C—P1C—C3C—C4C	−9.0 (2)	C17E—N12E—C10E—C9E	−178.1 (15)
C21C—P1C—C3C—C4C	−133.2 (2)	P1E—C9E—C10E—O11E	29 (3)
C9C—P1C—C3C—C4C	111.2 (2)	P1E—C9E—C10E—N12E	−136 (3)
O2C—P1C—C3C—C8C	172.05 (19)	C10E—N12E—C13E—C14E	87 (3)
C21C—P1C—C3C—C8C	47.9 (2)	C17E—N12E—C13E—C14E	−103 (2)
C9C—P1C—C3C—C8C	−67.7 (2)	N12E—C13E—C14E—C16E	44.0 (15)
C8C—C3C—C4C—C5C	0.7 (4)	N12E—C13E—C14E—C15E	169.2 (13)
P1C—C3C—C4C—C5C	−178.3 (2)	C10E—N12E—C17E—C18E	92 (3)
C3C—C4C—C5C—C6C	0.2 (4)	C13E—N12E—C17E—C18E	−78 (2)
C4C—C5C—C6C—C7C	−1.1 (4)	N12E—C17E—C18E—C19E	176 (2)
C5C—C6C—C7C—C8C	1.2 (4)	N12E—C17E—C18E—C20E	−60 (2)
C6C—C7C—C8C—C3C	−0.3 (4)	O2E—P1E—C21E—C22E	−58.1 (9)
C4C—C3C—C8C—C7C	−0.6 (4)	C3E—P1E—C21E—C22E	65.1 (8)
P1C—C3C—C8C—C7C	178.28 (19)	C9E—P1E—C21E—C22E	180.0 (8)
O2C—P1C—C9C—C10C	173.92 (17)	P1E—C21E—C22E—C23E	169.6 (7)
C21C—P1C—C9C—C10C	−62.3 (2)	C21E—C22E—C23E—C24E	171.2 (7)
C3C—P1C—C9C—C10C	52.5 (2)	C22E—C23E—C24E—C25E	167.3 (7)
C17C—N12C—C10C—O11C	1.6 (3)	C23E—C24E—C25E—C26E	60.5 (13)
C13C—N12C—C10C—O11C	177.2 (2)	C24E—C25E—C26E—C27E	171 (3)
C17C—N12C—C10C—C9C	−176.6 (2)	C25E—C26E—C27E—C28E	180 (4)

### Hydrogen-bond geometry (°)

**Table:**

*D*—H···*A*
—···
—···
—···
—···
—···
—···
—···
—···
—···
—···
—···
—···
—···
—···
—···
—···
—···
—···
—···
—···
—···
